# XcepFusion for brain tumor detection using a hybrid transfer learning framework with layer pruning and freezing

**DOI:** 10.1038/s41598-025-33970-z

**Published:** 2025-12-30

**Authors:** Deependra Rastogi, Prashant Johri, Seifedine Kadry, SeongKi Kim, Lalit Kumar, Vishwadeepak Singh Baghela, Arfat Ahmad Khan

**Affiliations:** 1 School of Computer Science and Engineering, IILM University, Greater Noida, Uttar Pradesh 201306 India; 2 School of Computer Science and Engineering, Galgotias University, Greater Noida, Uttar Pradesh 203201 India; 3 Department of Computer Science and Mathematics, Lebanese American University, Beirut, Lebanon; 4 Department of Computer Engineering, Chosun University, Gwangju, 61452 Republic of Korea; 5 Institute of Well-Aging Medicare & CSU G-LAMP Project Group, Chosun University, Gwangju, 61452 Republic of Korea; 6 Department of Computer Science, College of Computing, Khon Kaen University, Khon Kaen, 40002 Thailand

**Keywords:** Brain tumor, Magnetic resonance imaging, Convolutional neural network, Transfer learning, Deep learning, Xception, classification, Machine learning technique, Feature extraction, SVM, KNN, Decision tree, random forest, Logistic regression, Cancer, Computational biology and bioinformatics, Engineering, Health care, Mathematics and computing, Medical research

## Abstract

For effective treatment options and better patient outcomes, early and accurate diagnosis of brain tumors is essential. This research introduces an innovative strategy to improving brain tumor diagnosis accuracy by combining deep learning with traditional machine learning classifiers. This research investigation employs the Xception Convolutional Neural Network (CNN) through a transfer learning approach as a feature extractor via two distinct strategies: (1) pruning the CNN’s classification layers while freezing the remaining layers, and (2) utilizing feature extraction with all CNN layers frozen. The extracted features are subsequently classified utilizing five traditional classifiers: Support Vector Machine (SVM), Decision Tree (DT), K-Nearest Neighbors (KNN), Random Forest (RF), and Logistic Regression (LR). The suggested approaches are assessed using the BR35H: Brain Tumor Detection 2020 dataset, which is publicly accessible on Kaggle and includes a thorough collection of labeled MRI scans of the brain for both training and testing purposes. Results show that the hybrid models achieve exceptional performance, with both transfer learning-based strategies providing highly accurate tumor classification. Specifically, the Xception model with frozen CNN layers and feature extraction yielded testing accuracies of 0.9900 for Logistic Regression (LR) and 0.9850 for K-Nearest Neighbors (KNN). In comparison, pruning the CNN layers and freezing the remaining layers also resulted in comparable high performance, with testing accuracies of 0.9883 for KNN and 0.9900 for Logistic Regression (LR). According to these results, brain tumor diagnosis may be made much more efficient and accurate by combining deep learning feature extraction with standard machine learning classifiers.

## Introduction

With high rates of death and morbidity across the globe, brain tumours rank among the most serious health issues facing contemporary medicine^1,2^. Both benign and malignant forms of these aberrant cell proliferations in the brain are possible, but the latter are particularly dangerous because of their quick development and ability to infiltrate healthy tissues^3^. Recent research indicates that primary brain and central nervous system (CNS) tumours are becoming more common worldwide, which emphasises the significance of creating effective and precise diagnostic methods^4,5^. Magnetic Resonance Imaging (MRI) is often regarded as the most effective technique for identifying brain tumours due to its remarkable contrast resolution and ability to detect complex anatomical characteristics. However, accurately interpreting MRI images is a difficult process that greatly depends on the knowledge of radiologists. Manual diagnosis can be laborious and prone to mistakes due to inter-observer variability, visual fatigue, and human perception limits, particularly when differentiating distinct tumours that share similar visual features. These difficulties emphasise the necessity of automated, impartial, and repeatable diagnostic instruments.

Artificial intelligence (AI), especially machine learning and deep learning, has transformed the discipline of medical imaging. Of the several deep neural network models, Convolutional Neural Networks (CNNs) stand out for their handling of visual input. Convolutional neural networks are superior to conventional, hand-crafted feature extraction techniques in many image classification applications because they are able to learn hierarchical features from unprocessed image data^6,7^. On the other hand, especially when applied to well specified feature sets, classic machine learning classifiers—such as Support Vector Machines (SVM), Random Forests, and Logistic Regression—are acknowledged for their clarity and dependability. Improving the accuracy, efficiency, and generalisability of automated brain tumour detection systems by means of the combination of CNN feature learning skills with standard ML classifier decision-making strengths offers a convincing strategy.

The development of artificial intelligence (AI) has transformed the medical imaging industry especially in machin Although CNNs have shown progress in the field of medical image classification, their direct use for brain tumour diagnosis presents some difficulties. First of all, privacy concerns and the difficulties finding captioned medical photographs sometimes limit the expansion of medical databases^8,9^. To avoid overfitting and guarantee adequate generalisation across different tumour types and imaging settings, convolutional neural networks often require large quantities of labelled data^10,11^. Moreover, in clinical environments, where reasonable choices are crucial for developing confidence and enabling acceptance, CNN models’ clarity remains a major concern.

Though typically more interpretable and less keen on data, traditional machine learning classifiers cannot independently extract spatial and hierarchical imagine characteristics. They depend on either externally derived characteristics or manually created ones, which might not be sufficient to capture the intricate patterns shown in brain tumour photos. Thus, in performance and robustness, a solely CNN-based or ML-based method might be inadequate. A hybrid model combining the best of both worlds—deep CNN-based feature extraction and conventional ML-based classification—is needed to solve these constraints.

This work proposes a hybrid framework combining the Xception Convolutional Neural Network’s (CNN) strong feature extraction capabilities as the basic model with a method based on transfer learning and various traditional ML classifiers in order to enable reliable and exact identification of brain tumours and feature extraction. The Xception architecture is selected because of its use of depthwise separable convolutions, which enable effective computation and capture of rich and varied characteristics from medical images—a necessary ability when handling the complexity of MRI scans. Starting with transfer learning and using the pretrained Xception model first trained on the vast ImageNet collection, the proposed approach All convolutional layers of the model are maintained in a fixed state in order to maintain the integrity of the obtained visual characteristics and remove the need for considerable retraining. Eliminated are the higher classification layers, designed for the first training job. Instead, concentrate on preserving and freezing an intermediary layer, identified for its capacity to encode high-level, abstract information very useful for medical imaging analysis. Each MRI scan is encapsulated in the deep feature vector derived from this layer.

Combining knowledge and deep learning. Among the several kinds of deep neural network models, CNNs stand out for their visual input handling ability. Convolutional neural networks have a leg up in many image categorisation activities as they can learn hierarchical features from raw picture data, unlike conventional, manually-crafted feature extraction techniques^6,7^. On the other hand, especially when used on well specified feature sets, classic machine learning classifiers—such as Support Vector Machines (SVM), Random Forests, and Logistic Regression—are acknowledged for their clarity and dependability. Improving the accuracy, efficiency, and generalisability of automated brain tumour detection systems by means of the combination of CNN feature learning skills with standard ML classifier decision-making strengths offers a convincing path.

These CNN-derived features are then used as inputs for a suite of classical ML classifiers. The selected classifiers—Support Vector Machine (SVM), K-Nearest Neighbors (KNN), Decision Tree, Random Forest, and Logistic Regression—are each trained to distinguish between different types of brain tumors using the extracted feature vectors. The proposed framework combines the deep feature learning capabilities of CNNs with the structured decision-making power of ML classifiers, resulting in several advantages. This approach captures essential spatial abstractions vital for medical diagnosis, boosts generalization and robustness across various datasets, and enhances model interpretability, thereby making the system more adaptable and reliable for clinical applications.

The following contributions are included in this study:

- Utilizing Xception Convolutional Neural Networks (CNNs), with all convolutional layers frozen enables the model to leverage deep, hierarchical features learned from large-scale datasets like ImageNet. This preserves general visual representations that are effective for various image classification tasks.
- Trimming the fully connected classification layers of the CNN and feeding the output into conventional machine learning classifiers like Support Vector Machines and Random Forests enhances model generalization. These classifiers typically demonstrate superior performance with high-dimensional feature vectors and exhibit a reduced tendency to overfit, particularly in scenarios where training data is scarce.
- Freezing all CNN layers and removing the dense classification head reduces computational load, resulting in a lighter and more resource-efficient model.

The structure of this paper is outlined as follows. Section “Related work” examines current studies on brain tumor detection, emphasizing the difficulties that motivate the suggested hybrid approach. Section “Materials and visualization” outlines the datasets and visualizations employed, offering valuable insights into the characteristics of brain tumors. Section “Dataset description” details the image pre-processing techniques utilized to ready the data for the model. The fifth section delineates the hybrid methodology that integrates CNN feature extraction with various machine learning classifiers. Section “Augmentation and data generator” outlines the criteria for evaluating the performance of the model. Section Normalization step details the experimental findings, juxtaposing the proposed method against current methodologies. Section “Methodology adapted” presents the findings, examining the strengths, weaknesses, and practical implications. In conclusion, Sect. “Xception model as a base model” provides an outline of the study’s contributions and offers recommendations for future inquiries.

## Related work

Khan et al.^12^ introduced a multimodal classification approach that effectively combines deep learning models with robust feature selection strategies to enhance brain tumor detection. Utilizing a combination of MRI modalities such as T1, T2, and FLAIR, the study harnessed the strengths of convolutional neural networks (CNNs) in learning spatial hierarchies of features from medical images. They supplemented these with a filter-based feature ranking approach, which selected the most discriminative features across modalities, ultimately boosting classification performance. Their model outperformed traditional ML techniques, demonstrating that integrating multimodal data not only enriches the input space but also significantly improves diagnostic accuracy. This framework supports radiologists by providing highly discriminative features for tumor identification.

Using explainable artificial intelligence techniques, Rasool and colleagues developed CNN-TumorNet, a deep convolutional neural network architecture^13^, to get accurate brain tumour classification. By including Grad-CAM into the model, the scientists enabled doctors to view how the model categorised photos; this helped them ascertain which areas of the brain images were most significant to the model’s results. In a healthcare context, where interpretability is extremely helpful, openness and faith in artificial intelligence systems are very vital. Reaching state-of- the-art accuracy in tumour type classification, the network passed all of the strict assessments using enormous MRI datasets. The paper emphasises the need of medical artificial intelligence solutions being both efficient and understandable.

Asiri et al. examined in their work^14^ CNN hyperparameter tuning’s impact on classification accuracy in brain tumour detection challenges. To maximise important model parameters like kernel size, number of layers, dropout rate, and activation functions, they investigated many tuning techniques including grid search and random search. Their tests revealed that optimal hyperparameters produce notable generalisation across datasets and performance gains. The work shows how a well-calibrated CNN may preserve accuracy on unseen data, therefore addressing one of the fundamental difficulties in DL—overfitting. This paper offers practitioners hoping to create strong CNN architectures for clinical use a comprehensive road map.

Sarkar et al.^15^ presented a hybrid paradigm for brain tumour categorisation. Deep features obtained from a pretrained convolutional neural network (CNN) named AlexNet form the basis of this approach. These characteristics subsequently feed SVM, k-NN, and random forest among other machine learning classifiers. This combination’s concept is to use CNN’s enhanced feature representation capabilities as well as the generalisability of conventional ML techniques. Their results lead support vector machines (SVMs) to be the most successful classifiers for different types of tumours. This work shows the feasibility of merging deep feature embeddings with lightweight ML classifiers in order to build efficient and cheap diagnostic tools.

Mathivanan et al. explored the utility of transfer learning in brain tumor detection using limited labeled MRI data^16^. By employing pre-trained CNN models like VGG16 and InceptionV3 and fine-tuning them on brain tumor datasets, they effectively addressed the common issue of data scarcity in medical imaging. The transfer learning models not only reduced training time but also enhanced classification performance due to their rich pre-learned features from large-scale image datasets. The study presents transfer learning as a highly practical and resource-efficient approach for medical applications where high-quality labeled data are difficult to obtain.

Nahiduzzaman and colleagues^17^ proposed a hybrid and explainable model for MRI-based brain tumor classification, combining convolutional neural networks with ensemble machine learning techniques. They incorporated SHAP (SHapley Additive exPlanations) and LIME (Local Interpretable Model-agnostic Explanations) to provide insight into the model’s predictions. Their system was validated on multiple datasets and outperformed baseline methods in accuracy and interpretability. The integration of explainable AI ensures that clinicians can trace back the AI’s decisions, adding a layer of reliability necessary for real-world deployment.

This work^18^ by Bhimavarapu et al. created a two-phase brain tumour analysis pipeline comprising an initial segmentation stage using enhanced unsupervised clustering, then classification using a supervised ML model. By lowering non-informative background data, the unsupervised step separated tumour areas more efficiently, thereby improving the accuracy of the next classification job. Their approach showed good results in multiclass tumour classification and proved that exact presegmentation may greatly improve diagnosis results.

To improve MRI-based brain tumour classification, Mohanty et al. presented an original CNN model enhanced with soft attention mechanisms^19^. Dynamic emphasis of the attention layers on tumor-relevant areas of the input pictures enables the network to provide top priority for significant spatial information during training. Higher accuracy and more strong model predictions resulted from this approach addressing problems including background clutter and intra-class volatility. The soft attention component further enhanced interpretability by pointing out which picture portions were most important for categorisation.

Agarwal et al.^20^ developed a unique CNN architecture with convolutional and pooling operations especially for MRI scan-based brain tumour identification. They brought optimisation in layer connection and filter use, thereby enhancing feature abstraction capacity and lowering overfitting. On benchmark datasets, its design was assessed for good sensitivity and specificity in tumour area identification. Emphasising the need of model modification for clinical relevance, this study helps to create customised CNN architectures for medical imaging uses.

Basthikodi et al. concentrated on improving multiclass brain tumour diagnosis by means of a hybrid approach combining sophisticated feature extraction techniques^21^ with Support Vector Machine (SVM) classifiers. To raise MRI image quality, their work built a strong preprocessing pipeline comprising skull stripping, noise filtering, and contrast enhancement. Gray-Level Co-occurrence Matrix (GLCM) and Local Binary Pattern (LBP) were used for feature extraction to grab picture texture and spatial patterns. These characteristics loaded into an SVM for classification produced better accuracy over several tumour types. The methodological rigidity of the study and its focus on handmade elements underline its relevance to effective and interpretable ML-driven diagnostics.

Using MRI data, Saeedi and colleagues^22^ carried a comparison research combining convolutional neural networks (CNNs) with conventional machine learning models to identify brain tumours. Beginning with data augmentation and normalisation, they used a multi-stage pipeline to feature learn using a CNN then classify using models like SVM, Decision Trees, and k-NN. Their findings showed that while hybrid models achieved the optimal balance between accuracy and computational economy, CNNs shone in capturing spatial hierarchies. This method emphasises, especially in clinical settings with limited resources, the pragmatic advantage of combining DL and ML techniques.

Emphasising the importance of model tweaking for clinical use, Sadr et al. developed a deep learning model for precise brain cancer classification from MRI data. Designed^23^ as a convolutional neural network (CNN) trained using enhanced datasets in order to solve class imbalance. Using layer-wise tuning in concert with batch normalisation and dropout, their approach prevented overfitting. Under tests on pituitary tumours, meningioma, and glioma, the model outperformed the human one. By assessing the model over several datasets, they also demonstrated its durability and generalisability. This approach considerably increases the validity of DL models in medical diagnosis.

In their work^24^, Wageh et al. offered a complete system including traditional ML classifiers together with deep feature extraction and genetic algorithm-driven feature selection. Deep features were obtained using a pre-trained CNN and then optimised using a genetic algorithm to minimise feature dimensionality while maintaining discriminating ability. Using random forests and support vector machines among other machine learning models, these characteristics were categorised. While simultaneously reducing computing requirements, the approach shown notable improvements in classification accuracy. Using evolutionary algorithms for feature selection presents a creative way to increase the accuracy of diagnosis.

Kang and colleagues created an ensemble framework^25^ combining various CNN architectures and deep features collected from MRI images. Aggregated and supplied into ensemble classifiers including voting and gradient boosting classifiers, these elements Their approach underlined the complimentary character of deep features from many CNNs, which when aggregated greatly enhanced classification resilience. Showcasing its promise for real-world deployment where model consistency is crucial, the ensemble technique reduced model variation and improved diagnostic accuracy.

Using MRI images, Asiri et al. investigated a dual-network architecture^26^ integrating ResNet50 with U-Net to concurrently identify and categorise brain tumours. After localising the tumour area, the U-Net segmenter fed the fine-tunized ResNet50 for classification. The technique was evaluated for segmentation as well as classification using TCGA-LGG and TCIA datasets. Focussing categorisation on tumor-specific areas helped this integrated design not only raise classification accuracy but also enhance interpretability. The work distinguishes itself with its full-pipeline approach, which simplifies tumour detection and localisation.

Dixon and colleagues put up a hybrid learning architecture^27^ combining CNN-based learning with manual feature extraction. Their solution uses CNN-learned representations combined with radiomics-inspired elements, which are then classified using a fully connected neural network. The hybridisation sought to grab deep-learned patterns as well as domain knowledge. Results revealed improved tumour classification over several datasets, therefore stressing the synergy between traditional image analysis and deep learning methods in challenging diagnosis problems.

Examining interpretability in deep learning models for brain tumour diagnosis, Nhlapho et al. addressed the black-box question in artificial intelligence. On CNN architectures used on MRI data, they employed^28^ a set of interpretability methods including Grad-CAM and SHAP. The study assessed how various layers support final predictions and how doctors may profit from such understanding. The work significantly advances explainable artificial intelligence and opens the path for more honest and open medical artificial intelligence uses.

Semwal et al. developed a hybrid approach^29^ optimising a convolutional neural network with a support vector machine (CNN-SVM) by use of particle swarm optimisation (PSO). While the SVM supplied classification, the CNN managed feature extraction; PSO tuned hyperparameters for maximum accuracy. Their method improved tumour subtype classification and addressed overfitting and feature redundancy. A potential approach for precision diagnostics is provided by this new integration of evolutionary optimisation with hybrid modelling. To automatically detect brain tumours from radiological pictures, Natha and colleagues^30^ built a multi-model ensemble deep learning system. To increase resilience and lower model bias, they aggregated the results of many CNNs using a majority voting system. Tested over several datasets, the ensemble proved generalisability and low false positives. The capacity of the model to combine strengths of several architectures qualifies it as a trustworthy choice for implementation in varied clinical environments.

Based on the EfficientNet architecture tuned for speed and precision, Islam et al. presented BrainNet^31^, a brain tumour classification system. To improve convergence the model used sophisticated optimisation techniques like weight decay regularisation and learning rate warm-up. BrainNet exceeded traditional CNNs in accuracy and inference speed according evaluations using MRI datasets. Important for real-time clinical decision support systems, this study helps to create efficient artificial intelligence models. Remzan et al.^32^ presented a new ensemble learning-based feature extraction and categorisation system. They derived deep features using CNNs and used an ensemble system combining several ML classifiers. Their use of cross-validation driven feature selection—which guarantees just the most relevant features—was a major breakthrough. Emphasising the efficiency of ensemble approaches in reducing model variance and improving generalisation, their approach obtained good classification metrics over several datasets.

Raza and colleagues^33^ classified brain tumours from MRI images using transfer learning with DenseNet121. Changing the pre-trained model allowed the network to use the generic characteristics it had learnt before while acquiring domain-specific ones. The results confirmed that, particularly in cases with limited medical datasets, transfer learning has the ability to surpass models created from the ground up in general. The results stress the need of using pre-trained networks in contexts when annotated data availability is restricted.

Güler and Namlı^34^ investigated methods of classifier optimisation for DL-based brain tumour identification. They tweaked hyperparameters like learning rates, batch sizes, and dropout rates and evaluated many CNN designs. Training efficiency and classification accuracy were much raised by the optimisation approach. Their findings emphasises how crucial architectural choice and model tweaking are to get best performance in medical picture categorisation problems.

Liu et al. developed a CNN-based feature extraction approach^35^ coupled with traditional ML classifiers to classify MRI brain tumours. They found deep features extracted from a CNN following SVM, k-NN, and decision tree models. Their findings showed that hybrid DL-ML models might mix CNN’s high-level abstraction capacity with the interpretability and efficiency of traditional classifiers. This fusion model shows success in cases demanding both accuracy and explainability.

Malakouti et al.^36^ examined machine learning and transfer learning methods for brain tumour classification using MRI images. Combining pre-trained networks as ResNet and DenseNet with standard classifiers like SVM and XGBoost, they The models were evaluated both balanced and unbalanced using AUC and F1-score criteria. While traditional models offer interpretability and fast training, transfer learning performed very well on limited data. This dual point of view offers flexibility for different therapeutic needs.

Vimala et al.^37^ introduced a hybrid deep learning architecture integrating convolutional neural networks (CNNs) with recurrent neural networks (RNNs), thereby effectively collecting the spatial and temporal properties of MRI images particularly in long-term memory (LSTM) units. While the convolutional layers distinctly identified spatial properties across the image slices, the recurrent layers studied the evolution of spatial features over consecutive slices. Especially for infiltrative gliomas, the dual-level processing allows the model to identify between tumour grades with higher accuracy. The work reveals how effectively hybrid architectures enable to grasp intricate patterns in volumetric medical imaging data.

Based on an in-depth review of the existing literature, the following research gaps have been identified:

- There is a lack of systematic evaluation of feature fusion and selection strategies that can best leverage CNN-extracted representations for traditional ML classifiers.
- There is a need to enhance domain generalization and robustness in hybrid CNN-ML pipelines through techniques such as domain adaptation or dataset-agnostic feature learning.
- Few studies utilize or analyse the explainability potential of combining CNN feature extraction with interpretable classifiers to assist clinical decision-making.
- There is a need for standardized evaluation protocols and benchmark datasets to assess the robustness, efficiency, and scalability of hybrid CNN-ML frameworks.
- There is a lack of focus on designing lightweight and fast CNN-ML models suitable for real-time use in resource-constrained healthcare settings.

In response to the identified research gaps, this study explored a strategy of a robust and interpretable hybrid framework that leverages convolutional neural network (CNN) features in combination with traditional machine learning (ML) classifiers for brain tumor detection. The study will systematically evaluate various feature fusion and selection strategies to determine the most effective methods for optimizing CNN-extracted features for ML classification. Table 1 is showing the literature details.

**Table 1:** Literature Review.

Ref	Model Type	Method / Model Used	Dataset	Innovation	Strength	Research Gap
^12^	Hybrid DL + ML	CNN + SVM/RF with multimodal data	BRATS	Combined multimodal MRI features with robust selection	High precision and reduced overfitting	Limited to small dataset; lacks real-time validation
^13^	CNN (Explainable)	CNN-TumorNet + XAI	Custom MRI	Integrates explainability into CNN	Enhances trust and interpretability	Requires clinical deployment and scalability testing
^14^	CNN	Optimized CNN	BRATS 2020	Automated hyperparameter tuning	High consistency across folds	Needs comparison with TL models
^15^	CNN + ML	AlexNet + SVM/KNN/RF	BRATS MRI	Combines DL features with ML classifiers	Better feature generalization	Feature fusion can be further optimized
^16^	Transfer Learning	ResNet, VGG16	BRATS, Kaggle	Uses TL for MRI with fine-tuning	High accuracy with low training cost	Dataset imbalance not addressed
^17^	Hybrid DL + ML	CNN + XAI + ML	BRATS, TCIA	Introduces explainability with hybrid fusion	Very high accuracy and interpretability	High computational cost
^18^	ML (Unsupervised + SVM)	K-means + SVM	Custom MRI	Combines segmentation and ML classification	Improved edge detection	Not end-to-end DL; lacks automation
^19^	CNN + Attention	CNN + Soft Attention	Kaggle MRI	Attention mechanism for ROI focus	Better tumor localization	High model complexity
^20^	CNN	CNN	BRATS, Harvard Dataverse	Improved CNN architecture	Robust detection accuracy	Limited explainability
^21^	ML	SVM + Novel Feature Extraction	BRATS 2021	Unique handcrafted features	Simple and interpretable	Lower performance than CNN
^22^	Hybrid	CNN + Random Forest	Kaggle MRI	Combines CNN and ML	Balanced accuracy and efficiency	Needs end-to-end optimization
^23^	CNN	CNN	BRATS 2020	End-to-end automated CNN pipeline	Robust classification	No interpretability layer
^24^	Hybrid DL + ML	CNN + SVM + Genetic Algorithm	BRATS 2020	Genetic feature selection	High efficiency	Computationally heavy
^25^	Ensemble DL + ML	CNN Ensemble + SVM/RF	BRATS 2018	Feature-level ensemble	Improved robustness	Needs real-time evaluation
^26^	CNN + Segmentation	ResNet50 + U-Net	TCGA-LGG, TCIA	Combines classification & segmentation	Superior localization & accuracy	High GPU requirements
^27^	Hybrid DL + ML	CNN + ML	BRATS 2021	Fuses ML with DL for optimization	Reduced complexity	Limited transferability
^28^	CNN (Explainable)	Explainable CNN + Grad-CAM	BRATS 2020	Visual interpretability integration	High clinical potential	Lower accuracy vs. non-XAI models
^29^	Hybrid	CNN-SVM + PSO	Kaggle MRI	PSO optimization for feature weights	Improved hybrid performance	Limited generalization
^30^	Ensemble DL	VGG + ResNet + DenseNet	BRATS	Multi-architecture ensemble	Exceptional classification accuracy	High computational cost
^31^	CNN	EfficientNet	Custom MRI	EfficientNet optimization	Lightweight and accurate	Needs more diverse data
^32^	Ensemble DL + ML	CNN + Ensemble ML	BRATS 2019	Combined multiple feature sources	Enhanced model stability	Lacks real-time validation
^33^	Transfer Learning	DenseNet121	BRATS, TCGA	Transfer learning with DenseNet	Accurate on small data	Model explainability missing
^34^	CNN	CNN + Optimized Parameters	BRATS	Classifier optimization	Improved efficiency	Dataset generalization needed
^35^	Hybrid DL + ML	CNN + SVM/KNN/RF	BRATS 2021	CNN feature extraction + ML classification	Easy to interpret	Lower accuracy than TL models
^36^	Transfer Learning	CNN + TL + SVM	BRATS	Combined TL and ML techniques	Improved adaptability	Limited dataset variation
^37^	CNN + GDD	Deep Learning + GDD	Kaggle MRI	Introduces GDD feature approximation	Accurate classification & survival prediction	Complex preprocessing stage

## Materials and visualization

### Dataset description

**Fig. 1:**
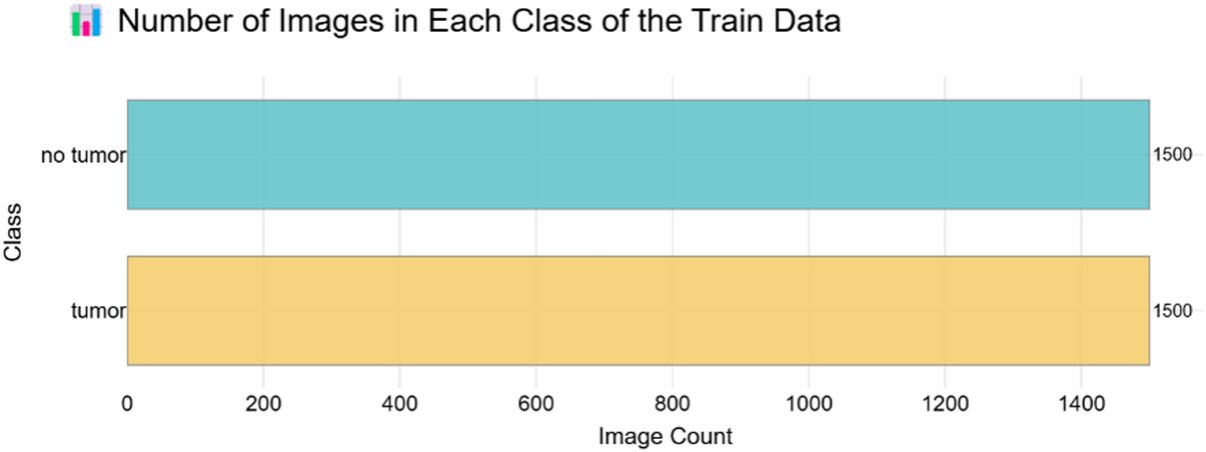
Number of images in each class of the train data.

Specifically concentrating on the diagnosis of brain tumours using MRI (Magnetic Resonance imaging) images, the Br35H: Brain Tumour diagnosis Dataset is a freely accessible dataset carefully curated to promote research and development in medical image processing. In binary classification problems, where the objective is to differentiate MRI images with brain tumours from those free of them, this dataset is absolutely vital. There are 3,000 brain MRI scans overall, split into two categories: “yes”, or images showing a tumour; “no”, or images lacking a tumour [Figure 1; Table 2]. The collection offers routinely used image formats together with high-resolution T1-weighted MRI pictures. Usually greyscale, these photos may have RGB representations depending on the preprocessing techniques applied by contributors. Though their dimensions differ, the photos in the dataset are generally scaled to consistent forms like 128 × 128 or 224 × 224 pixels for model training needs. Images from the “yes” class clearly show one or more tumours with variable forms, sizes, locations, and intensities, therefore guiding models to identify various patterns of aberrant tissue development. Conversely, the “no” class consists of normal MRI images devoid of any obvious tumour, thereby offering a necessary baseline for differentiating healthy from sick brain tissue. Separate folders for these classes help to make the dataset easily available and useable for machine learning applications. Although the Br35H dataset is strong for classification, its applicability for exact tumour border identification is limited until reinforced with hand annotations as it lacks segmentation masks. Along with basic preprocessing tasks include picture resizing, pixel value normalizing—usually ranging from 0 to 255 to 0–1—and cautious train-test splits to prevent data leaking.

**Table 2:** Number of images belonging to two Classes.

Tumor class	Denoted label	Number of MRI images
Tumor	1	1500
No Tumor	0	1500

Figuring out the dataset into discrete subsets for the goals of training and testing is a regular and necessary habit in deep learning systems [Table 3]. This approach involves separating the input features—such as pre-processed MRI scans used as the model’s learning input—from their matching labels, which denote the expected classification outcomes (e.g., cancer or not). 80% of the data is usually set aside for the training set, which helps the model to be fit and helps to adjust its parameters using learning methods. The last 20% is kept aside as the test set, intended to evaluate the model’s capacity for objective generalisation to fresh, untested data.

**Table 3:** Distribution of Dataset.

Tumor set	Number of MRI images
Training	2400
Testing	600

This partitioning is performed using a randomized sampling mechanism to ensure that the data distribution remains statistically representative across both subsets, thereby minimizing sampling bias. To ensure reproducibility and consistency of the results across different runs of the experiment, a fixed random seed (random state) is specified. This ensures that the random splitting process yields the same data partitioning each time the code is executed. Accurate train-test splitting is essential for mitigating overfitting and underfitting, and for generating reliable performance metrics. Table 4 is showing characteristics of the Dataset Used in This Study.

**Table 4:** Characteristics of the dataset used in this Study.

Attribute	Description
Dataset name	BR35H: Brain Tumor Detection 2020
Type of data	MRI Brain Images (T1-weighted contrast-enhanced)
Total number of images	3,000 images
Categories/Classes	Two classes: Tumor and No Tumor
Image resolution	224 × 224 pixels (after preprocessing / resizing)
Data format	JPEG / PNG
Color scale	RGB (converted from grayscale during preprocessing)
Annotation type	Label-based (classified as Tumor or No Tumor)
Data Split used in study	80% Training, 15% Testing
Acquisition modality	Magnetic Resonance Imaging (MRI)
Purpose	Used to train and evaluate deep learning models for brain tumor detection and classification

## Pre-processing step

### Augmentation and data generator

The purpose of this section is to provide a computationally efficient and technically sound envision pre-processing and augmentation pipeline for use in medical image investigation, particularly MRI for the purpose of detecting brain tumors^38^. In order to do real-time picture augmentation and pre-processing while training, the implementation makes use of the ImageDataGenerator class from Keras. By subjecting the input photos to a sequence of controlled, random modifications, data augmentation creates the illusion of a larger and more diverse training dataset^38^. Particularly helpful in medical imaging, where expert annotation makes the acquisition of huge labeled datasets time-consuming and expensive. By mimicking a range of real-world circumstances, augmentation helps reduce the risk of overfitting, strengthens the model, and enhances its capability to generalize. During training, the pipeline makes sure the model sees a wide variety of enhanced picture samples, which improves its capacity to generalize to new data. Normalizing pixel values is the first step in the preparation phase. Since MRI images are typically stored with 8-bit intensity values in the range [0, 255], the normalization operation rescales pixel intensities to the range [0.0, 1.0], using the transformation:

$$\:{x}_{normalized}=\frac{{x}_{original}}{255}$$

This normalization step is critical for ensuring numerical stability and accelerating the convergence of deep neural networks by homogenizing the input distribution. Following normalization, various augmentation strategies are employed to synthetically increase the size and diversity of the training data. Random image rotations are permitted up to ± 90◦, modelled using the 2D rotation matrix:

$$\left[ {\begin{array}{*{20}c} {x^{\prime } } \\ {y^{\prime } } \\ \end{array} } \right] = \left[ {\begin{array}{*{20}c} {\cos \theta } & { - \sin \theta } \\ {\sin \theta } & {\cos \theta } \\ \end{array} } \right]\left[ {\begin{array}{*{20}c} x \\ y \\ \end{array} } \right]$$

where θ is a randomly sampled angle. This transformation simulates different anatomical orientations, improving the model’s rotational invariance.

To simulate perspective variations, shear transformations are applied using a shear factor λ. Horizontal shearing is given by the affine matrix:

$$\:\left[\begin{array}{c}{x}^{{\prime\:}}\\\:{y}^{{\prime\:}}\end{array}\right]=\left[\begin{array}{cc}1&\:{\uplambda\:}\\\:0&\:1\end{array}\right]\left[\begin{array}{c}x\\\:y\end{array}\right]=\left[\begin{array}{c}x+{\uplambda\:}\mathrm{y}\\\:y\end{array}\right]$$

Zoom augmentation is also implemented to mimic spatial scaling effects. This is represented mathematically by:

$$\:\left[\begin{array}{c}{x}^{{\prime\:}}\\\:{y}^{{\prime\:}}\end{array}\right]=\left[\begin{array}{cc}{s}_{x}&\:0\\\:0&\:{s}_{y}\end{array}\right]\left[\begin{array}{c}x\\\:y\end{array}\right]$$

where $\:{s}_{x}$ and $\:{s}_{y}$ are randomly chosen scaling factors. This permits the model to learn from features at manifold resolutions.

Flipping operations further enhance spatial invariance. Horizontal and vertical flips are represented by the matrices:

Horizontal Flip:

$$\:\left[\begin{array}{c}{x}^{{\prime\:}}\\\:{y}^{{\prime\:}}\end{array}\right]=\left[\begin{array}{cc}-1&\:0\\\:0&\:1\end{array}\right]\left[\begin{array}{c}x\\\:y\end{array}\right]$$

Vertical Flip:

$$\:\left[\begin{array}{c}{x}^{{\prime\:}}\\\:{y}^{{\prime\:}}\end{array}\right]=\left[\begin{array}{cc}1&\:0\\\:0&\:-1\end{array}\right]\left[\begin{array}{c}x\\\:y\end{array}\right]$$

These augmentation techniques collectively help the model become invariant to positional and structural variations in the input images, which is particularly valuable for detecting tumors of varying shape, size, and location.

To manage the input pipeline efficiently, the model utilizes data generators. In Keras, a data generator is an iterator that loads and pre-processes image data in real-time, yielding it in mini-batches during training. This approach is memory-efficient, as it eliminates the need to load the entire dataset into memory at once. It also supports dynamic augmentation, meaning that each epoch may see a different version of the same image, further enriching the diversity of the training process.

For validation and testing, a separate data generator is instantiated with only the normalization operation, ensuring that evaluation metrics are computed on original, unaltered images. The parameter validation_split = 0.2 internally reserves 20% of the dataset for validation, facilitating stratified sampling without the need for manual directory restructuring.

Image loading is handled via the flow_from_directory() method, which streams images directly from disk in real time. The images are resized to 224 × 224 pixels, consistent with the input dimensions required by widely used convolutional neural network architectures. Although MRI data are typically grayscale, color_mode=’rgb’ ensures compatibility with models pretrained on RGB datasets like ImageNet by replicating single-channel data across three channels.

To avoid overfitting, images are randomly shuffled (shuffle = True) before each training epoch. The batch size is set to 32 for training and 16 for validation. The labels are processed in categorical mode, meaning they are one-hot encoded as follows:

$$\:Tumor\:\left(class\:1\right):\:\left[1,\:0\right],\:\:NoTumor\:\left(class\:0\right)\::\:[0,\:1]$$

This encoding format is compatible with the categorical cross-entropy loss function:

$$\:{L}_{CCE}=-\sum\:_{i=1}^{C}{y}_{i}\mathrm{l}\mathrm{o}\mathrm{g}\left(\widehat{{y}_{i}}\right)$$

where C denotes the numeral of classes, $\:{y}_{i}\:$is the ground truth label, and $\:\widehat{{y}_{i}}\:$is the foreseen probability for class i.

### Normalization step

Normalising numerical input values within a predefined range helps to increase the accuracy and efficiency of the learning process^39^, hence it is among the most crucial preprocessing actions in deep learning. Working with picture data, where pixel values are sometimes expressed as integers within a range of 0 to 255, this procedure is very crucial. With 8-bit representation per colour channel, standard picture formats like PNG or JPEG contain pixel intensities; each pixel takes values from 0 (totally dark) to 255 (entirely brilliant).

**Fig. 2:**
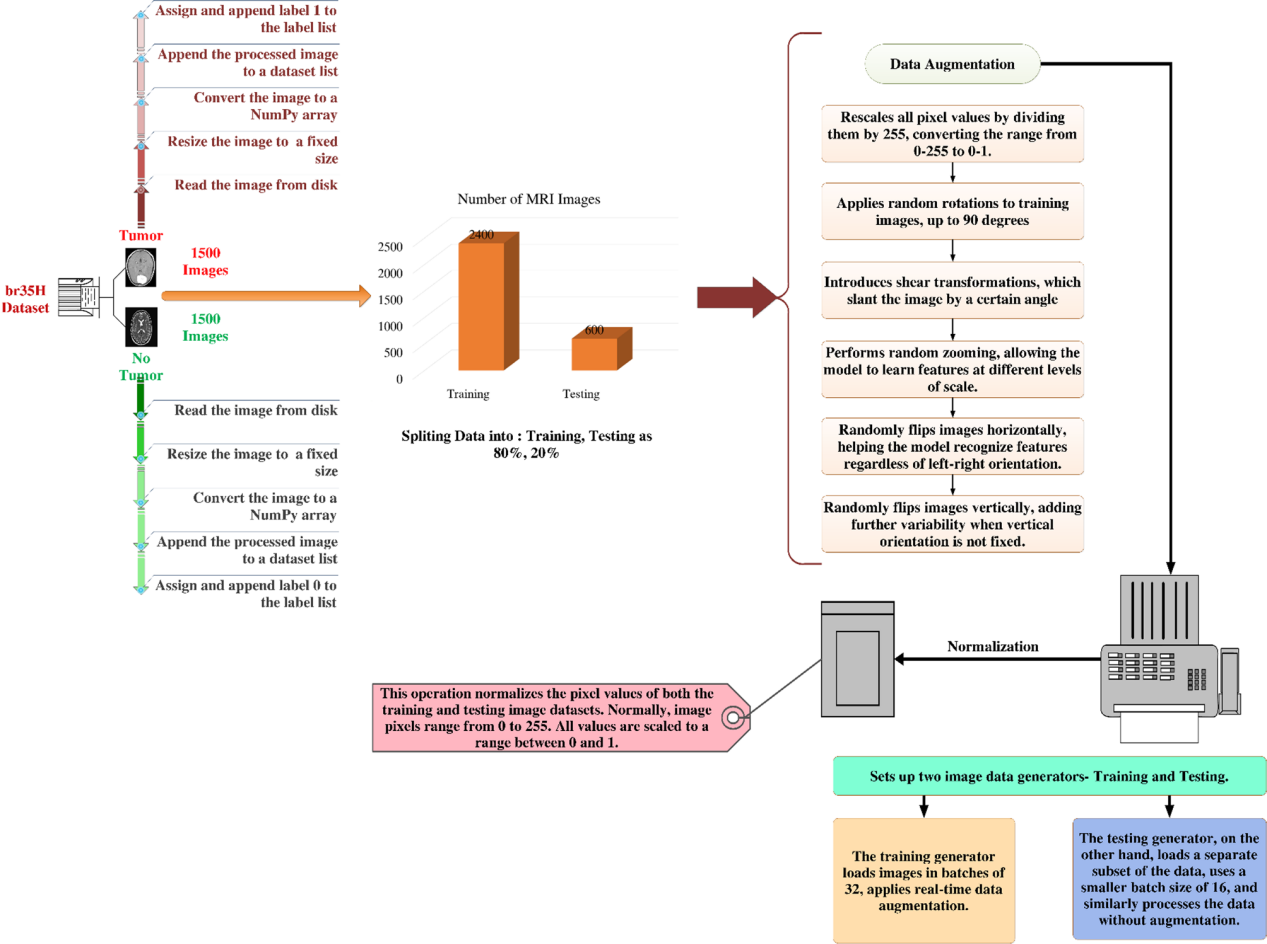
Pre-processing working flow.

Normalisation allows one to execute this pixel value transition to a floating-point range of 0.0 to 1.0 for numerous purposes. It first increases ML model efficiency by ensuring homogeneity and standardise of all input properties. By rescaling pixel values, make models more effective and thus shorten training time and speed up convergence. Gradient-based optimisation methods such as gradient descent considerably helps from this similarity in order to reduce the loss function during model training. Appropriate scaling of the input data helps to stabilise the optimisation process, thereby preventing problems such exploding or disappearing gradients, which could arise from either too big or too small input values for the activation functions of deep neural networks^39^. Normalising also helps to guarantee improved numerical stability during training as it helps to avoid unduly affecting the model by differences between input characteristics. This method improves model performance, accelerates convergence, and raises prediction accuracy by translating pixel values from integers within the 0–255 range into a floating-point range of 0.0 to 1.0. Therefore, normalisation is very important in the pre-processing pipeline especially for high-dimensional and complicated data, including medical imaging, where exact and reliable model training is absolutely vital. Figure 2 is shows the working flow of pre-processing.

Why Normalize the Data?

- Faster Convergence: Neural networks tend to converge faster when input data is normalized. When the pixel values are divided by 255, you transform the range of input data to a uniform scale between 0 and 1, which allows the model’s optimization algorithm (like gradient descent) to progress more smoothly. Large values can cause the optimization process to struggle, and smaller, more uniform values help avoid that.
- Improved Numerical Stability: In deep learning models, particularly those involving activation functions like sigmoid or tanh, large input values can lead to gradients that are too small or too large, causing problems such as exploding or vanishing gradients. By normalizing the data, we mitigate this risk and make the optimization process more stable.

- Consistent Input Across Features: When working with data that has different scales, such as images where pixel intensities might range from 0 to 255, it’s important to have consistency in the input range. By dividing by 255, we ensure that all pixel values fall in the same range, making the model more efficient and less likely to be biased toward certain features due to their range.

The normalization operation:

$$\:{X}_{train}=\frac{{X}_{train}}{255.0}$$

can be broken down as follows:

- $\:{X}_{train}\:$represents the pixel values of the training images.
- Each pixel value initially lies within the range [0, 255], where 0 represents the darkest possible pixel (black) and 255 represents the brightest (white).
- Dividing the pixel values by 255.0 scales them to the range [0.0, 1.0], where 0.0 is black and 1.0 is white. Intermediate values represent varying intensities of gray in a grayscale image, or different intensities for each of the RGB channels in a color image.

In other words:

$$\:New\:pixel\:value=\frac{Old\:pixel\:value}{255}$$

This operation applies to each pixel in every image in adapted dataset, effectively normalizing the entire image dataset.

## Methodology adapted

### Xception model as a base model

**Fig. 3:**
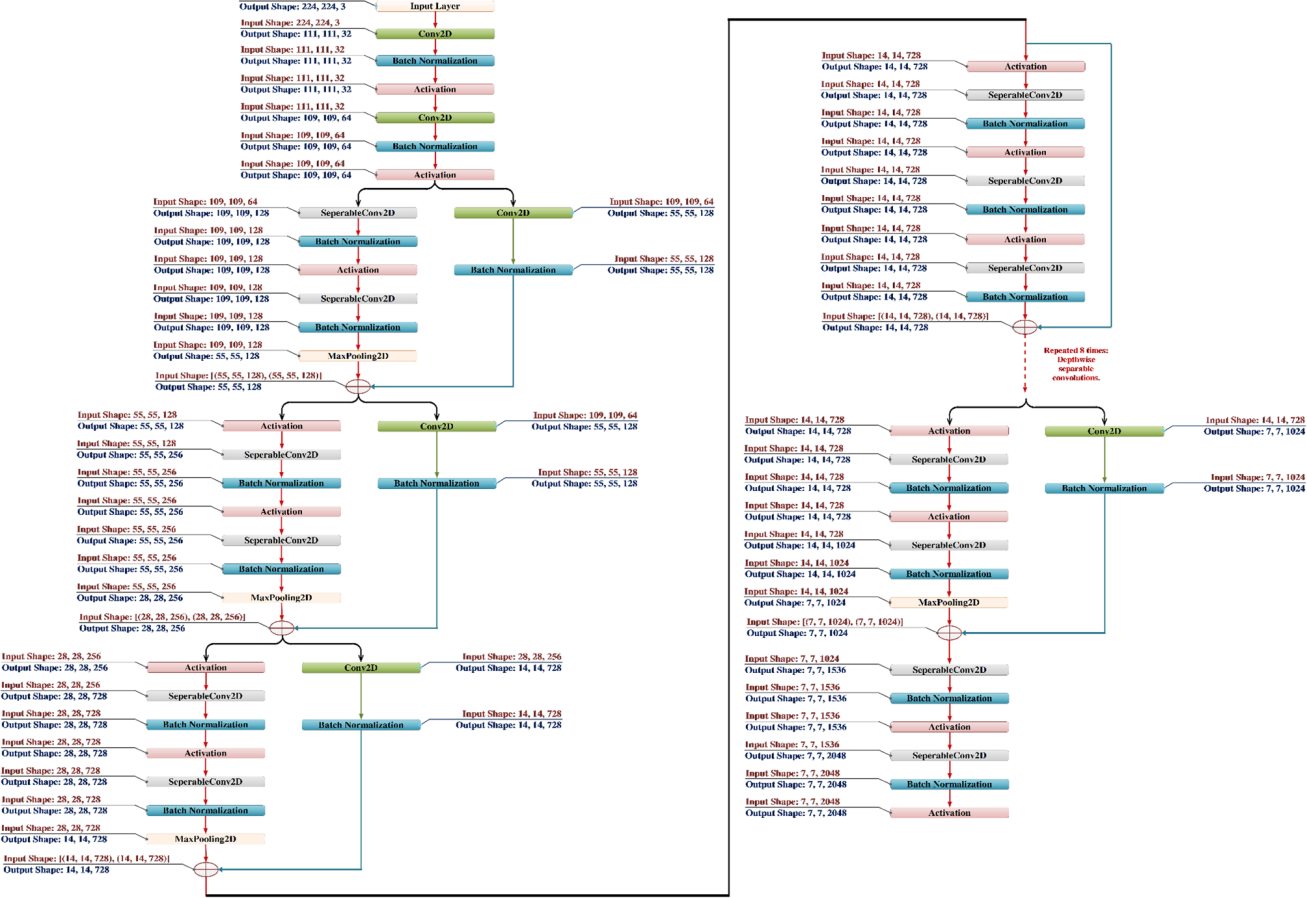
Xception Architecture Walkthrough.

Xception is a deep convolutional neural network architecture [Figure 3] introduced by Google researchers, which leverages depthwise separable convolutions to improve efficiency and performance over traditional convolutional approaches^40^. It can be viewed as an extension and refinement of the Inception architecture. Inception modules are designed to capture multi-scale features by applying multiple convolutional operations (e.g., 1 × 1, 3 × 3, 5 × 5 convolutions) in parallel—these parallel branches, often referred to as towers, are then concatenated to form a rich feature representation. This architecture effectively factorizes convolutions spatially and across channels, enabling deeper and more efficient networks^40^. The Xception architecture interprets the Inception module as an intermediary between ordinary convolutions and depthwise separable convolutions, therefore stretching this notion to an extreme. Originally, a depthwise convolution that performs spatial filtering on each input channel independently, followed by a pointwise convolution (1 × 1 convolution) that combines the results of the depthwise step across channels in a linear manner^41^, divides a standard convolution into two distinct steps. While keeping expressive ability, this decomposition significantly reduces processing costs and the number of parameters. Depthwise separable convolutions in this sense can be seen as Inception modules with several towers, where each input channel is processed separately before being combined back together. Inspired by this knowledge, Xception presents a novel architecture whereby depthwise separable convolutions completely replaces Inception modules, producing a model that is not only more efficient but also clearly more effective over several large-scale image classification benchmarks. By means of architectural simplicity, Xception can get exceptional performance while preserving or lowering computing complexity relative to past models such as Inception-v3^41^.

Xception (Extreme Inception) is a Convolutional Neural Network (CNN) architecture that rethinks convolutional blocks by fully decoupling spatial and channel-wise correlations—something traditional convolution layers entangle. This design is grounded in two principles:

- Depthwise Separable Convolution – Fully Decoupling Representation Learning.
- Residual Connections – Facilitating Deep Feature Learning.

#### Depthwise separable convolution - fully decoupling representation learning

In a standard 2D convolutional layer:

- You apply a set of 3D kernels (height × width × input_channels) to the input tensor.
- Each filter interacts with all channels simultaneously and learns both spatial features (patterns in height × width) and inter-channel dependencies in one operation.
- For an input of shape (H, W, $\:{C}_{in}$) and $\:{C}_{out}$ output channels, the computational cost is:

$$\:{Cost}_{standard}=H\times\:W\times\:{C}_{in}\times\:K\times\:K\times\:{C}_{out}$$

where K is the kernel size.

**Fig. 4:**
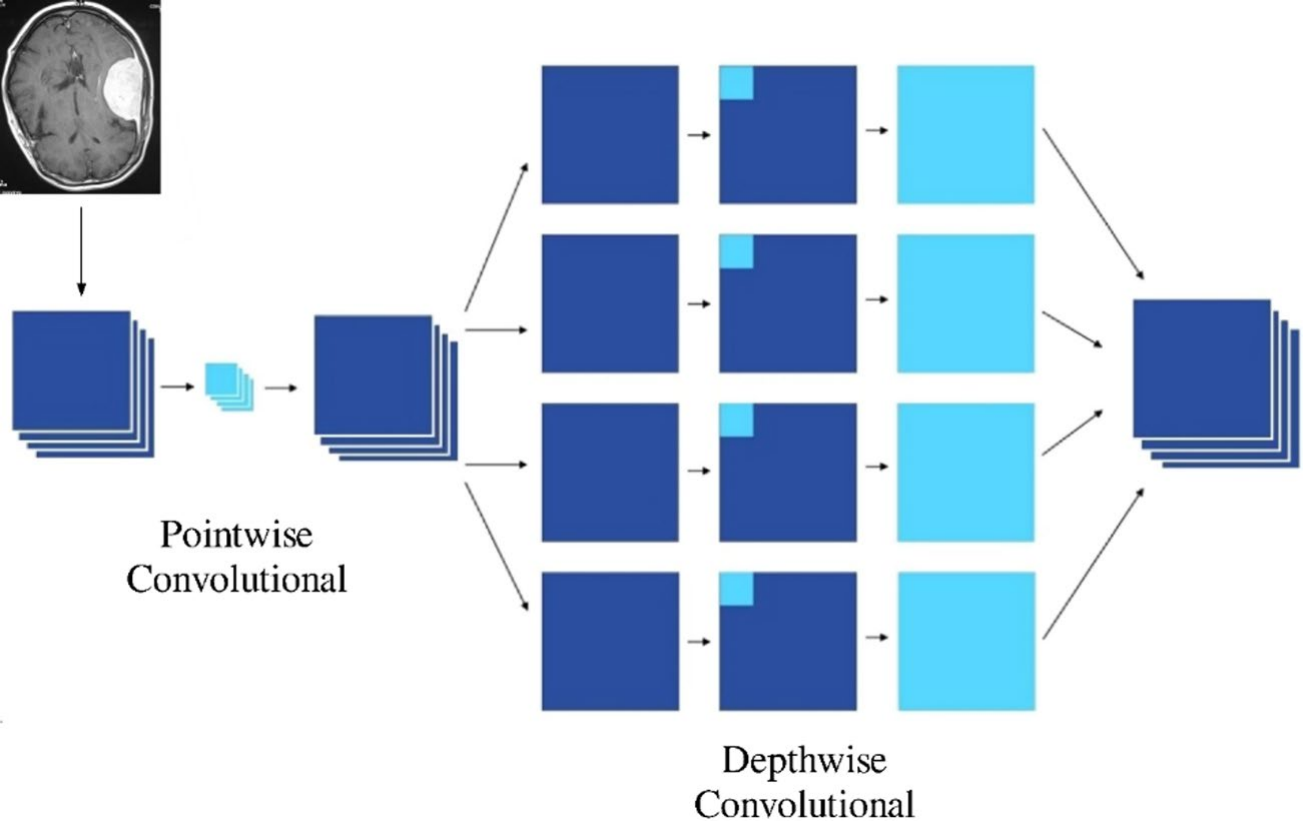
Pointwise and Depthwise working.

Xception’s Insight: François Chollet (creator of Xception) proposed that spatial and cross-channel correlations need not be learned jointly. Instead, they can be learned sequentially and independently—which is what depthwise separable convolutions do. Figure 4 shows the working of Pointwise and Depthwise Convolution.

1. Step 1: Depthwise Convolution.

- Applies one filter per input channel, not across all channels.
- Each filter is responsible only for spatial feature extraction.
- For $\:{C}_{in}$ channels, there are $\:{C}_{in}$ separate spatial filters.

The computational cost is:

$$\:{Cost}_{depthwise}=H\times\:W\times\:{C}_{in}\times\:K\times\:K$$

2. Step 2: Pointwise Convolution (1 × 1 Convolution).

- Applies a 1 × 1 convolution across the depth (channels).
- Learns inter-channel correlations by linearly combining the channel-wise outputs.
- Number of filters = output channels $\:{C}_{out}$.

The computational cost is:

$$\:{Cost}_{pointwise}=H\times\:W\times\:{C}_{in}\times\:{C}_{out}$$

3. Total Cost of Depthwise Separable Convolution

$$\:{Total}_{DSConv}=H\times\:W\times\:\left({C}_{in}\times\:{K}^{2}+\:{C}_{in}\times\:{C}_{out}\right)$$

This is significantly cheaper than standard convolution, especially when K = 3 and 541.

$$\:{C}_{in}$$ is large.

#### Residual connections – facilitating deep feature learning

Residual connections were introduced in ResNet to tackle the degradation problem, where deeper models tend to perform worse due to optimization difficulties.

In the Xception architecture:

- Each convolutional block is wrapped in a shortcut connection, defined as:

$$\:Output=F\left(x\right)+x$$

where F(x) is the output of a series of depthwise separable convolutions.

- If the input and output dimensions do not match (e.g., due to strided convolution or channel expansion), a 1 × 1 projection is applied to the shortcut to align their shapes before the addition.

### Integrated Xception model with transfer learning layers

In the proposed transfer learning architecture, a pretrained Xception model is integrated as a robust feature extractor. Xception, short for “Extreme Inception”, is a convolutional neural network trained on the ImageNet dataset, capable of extracting rich and semantically meaningful features from input images. The output of the Xception model yields a high-dimensional feature tensor of shape (7, 7, 2048), where each of the 2048 channels corresponds to an abstract feature map capturing spatial and contextual patterns.

The model architecture leverages depthwise separable convolutions, which drastically reduce computation while maintaining representational power. The total number of parameters in the Xception backbone is approximately 20, 861, 480. For transfer learning purposes, these parameters are typically frozen to preserve the pretrained feature representations and minimize overfitting on small target datasets.

After constructing the convolutional base, a 2D Global Average Pooling (GAP) layer is used to flatten the feature vector of length 2048 by reducing each 7 × 7 feature map to a single scalar value by average pooling. This method streamlines the design by omitting complex layers with many parameters, while also preserving the core of each feature map by activation summarization.

The GAP layer’s output is subsequently sent via a dense, fully linked layer with 64 hidden units to enable a lower-dimensional embedding. This layer’s number of trainable parameters is given by:

$$\:{Params}_{dense}=\left(2048\times\:64\right)+64=\mathrm{131,136}$$

where the first term corresponds to the weight matrix and the second term accounts for the bias vector. This transformation enables more effective learning of task-specific features.

A Dropout layer is added after the dense layer to avoid overfitting. To improve the model’s generalizability without adding more parameters, this regularization strategy randomly deactivates certain neurons during training.

A second dense layer with two output units, representing binary classification, makes up the last step of the design. This layer produces class probabilities based on the assumption of a softmax activation function. In this stratum, there are a total of:

$$\:{Params}_{output}=\left(64\times\:2\right)=130$$

The end-to-end forward propagation of the model can be mathematically expressed as:

$$\:\widehat{y}=softmax({W}_{2}.Dropout\left(\sigma\:\left({W}_{1}.GAP\left(F\left(x\right)\right)+{b}_{1}\right)\right)+{b}_{2})$$

where F(x) denotes the output feature map from the Xception base, W1 and W2 are the weight matrices of the two dense layers, b1 and b2 are the corresponding bias vectors, and σ represents a non-linear activation function, typically ReLU.

**Fig. 5:**
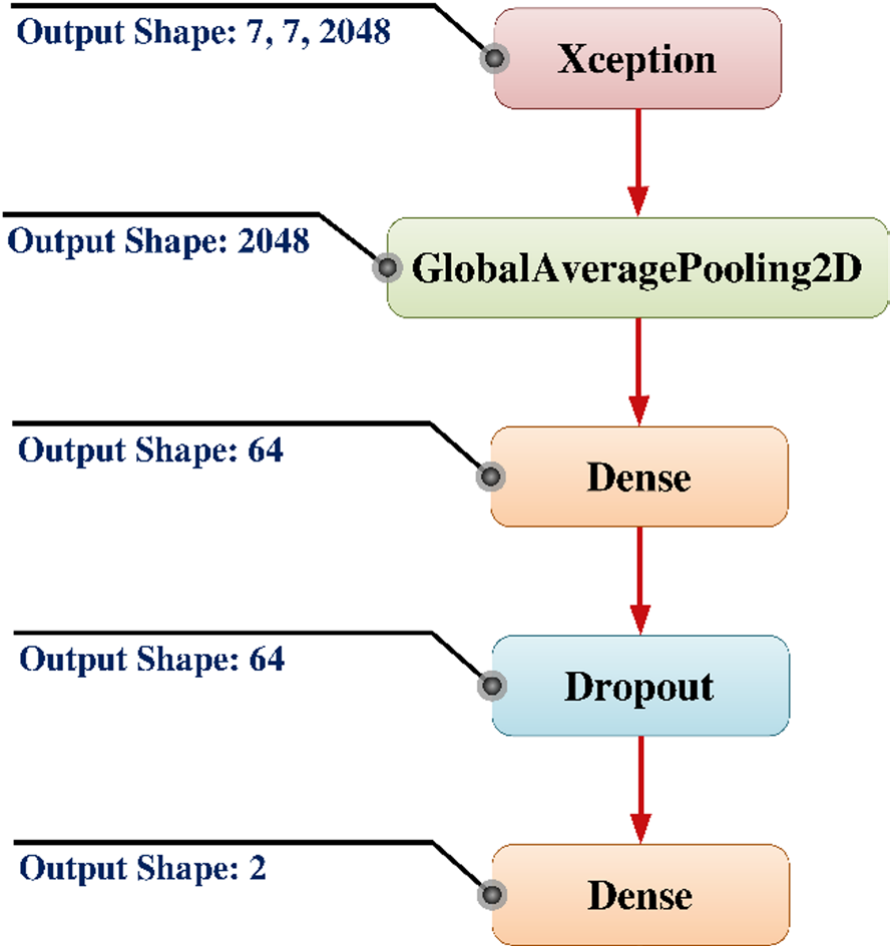
Integrated Xception Model with Transfer Learning Layers.

In summary, this architecture exemplifies an effective use of transfer learning by combining a powerful, pretrained convolutional base with a lightweight, task-specific classifier [Figure 5]. It enables rapid convergence, reduced overfitting, and improved accuracy on limited datasets by leveraging the generality of features learned from large-scale data.

### Feature extraction with a frozen all CNN layers

In this approach, a pre-trained Convolutional Neural Network (CNN) is adapted for feature extraction by freezing all of its layers. Let the CNN be parameterized by

$$\:\theta\:=\left\{{\theta\:}_{1},{\theta\:}_{2},{\theta\:}_{3},{\theta\:}_{4},\dots\:\dots\:\dots\:,{\theta\:}_{L}\right\}$$

where each $\:{\theta\:}_{i}$ represents the learnable weights of layer i, and L is the total number of layers in the network.

During training, weight updates are typically governed by gradient descent:

$$\:{\theta\:}_{i}\leftarrow\:{\theta\:}_{i}-\eta\:\bullet\:{\nabla\:}_{{\theta\:}_{i}}L\left(\theta\:\right)$$

where η is the learning rate and L is the loss function.

Freezing the layers implies:

$$\:{\nabla\:}_{{\theta\:}_{i}}L\left(\theta\:\right)=0\:\:\:\:\forall\:\:i\:\in\:\:\{1,\:2,\:.\:.\:.\:,\:L\}$$

This ensures that the pre-trained knowledge (typically learned from a large dataset such as ImageNet) is preserved and not overwritten during further training or inference. In implementation, this is achieved by marking all layers as non-trainable.

Next, instead of using the full CNN including the classification head—which generally consists of fully connected (dense) layers that map the learned features to a set of class probabilities—we discard this portion.

Mathematically, if the original CNN can be decomposed as:

$$\:CNN\left(X\right)=g\left(f\right(X\left)\right)$$

where f (X) denotes the feature extractor (the convolutional base) and g(·) denotes the classifier, we retain only f (X) for our purpose.

In this specific case, the feature extractor is taken to be the portion of the CNN up to the layer at index − 4, giving:

$$\:f\left(X\right)={CNN}_{\left[0:-4\right]}\left(X\right)$$

where $\:X\in\:{R}^{\mathrm{n}\times\:\mathrm{H}\times\:\mathrm{W}\times\:\mathrm{C}}$ represents a batch of n input images, each of spatial dimension H ×W with C color channels.

Passing the training and testing sets through f (X) yields high-dimensional feature tensors:

$$\:{F}_{train}=f\left({X}_{train}\right)\in\:{R}^{{(n}_{train}\times\:h\times\:w\times\:d)}$$

$$\:{F}_{test}=f\left({X}_{test}\right)\in\:{R}^{({n}_{test}\times\:h\times\:w\times\:d)}$$

Here, h, w, d represent the spatial dimensions and depth (i.e., number of channels) of the extracted feature maps from the selected CNN layer.

Since most classical machine learning models (e.g., Support Vector Machines, Logistic Regression, Random Forests) expect 2D input matrices of shape $\:{R}^{n\times\:m}$, the 3D feature maps for each image must be flattened. This transformation is applied as:

$$\:{\stackrel{\sim}{F}}_{train}=reshape\left({F}_{train}\right)\in\:{R}^{{n}_{train}\times\:(h\bullet\:w\bullet\:d)}$$

$$\:{\stackrel{\sim}{F}}_{test}=reshape\left({F}_{test}\right)\in\:{R}^{{n}_{test}\times\:(h\bullet\:w\bullet\:d)}$$

Thus, each image is now represented by a single feature vector of length:

$$m = h \cdot w \cdot d$$

capturing hierarchical spatial features learned by the CNN.s.

### Pruning CNN classification layers and freezing remaining layer

In this method, a pre-trained CNN is adjusted by freezing the convolutional layers that remain after manually eliminating the classification layers. Then, the network is used as a fixed feature extractor. Reducing the size of input photos so they may be utilized in traditional ML models is the main objective. Layers for classification, which usually make up the final few of a convolutional neural network (CNN), include:

- Global average pooling.
- Fully connected (dense) layers.
- Dropout layers.
- Output layer (e.g., softmax or sigmoid).

The code sequentially removes the last four layers from the CNN. Mathematically, we can represent the original CNN as a function:

$$\:CNN\left(X\right)=g\left(f\right(X\left)\right)$$

where $\:f\left(X\right)$ is the convolutional base (feature extractor) and $\:g\left(f\right(X\left)\right)$ is the classifier (fully connected layers). By removing the classification layers $\:g\left(f\right(X\left)\right)$, we isolate $\:f\left(X\right)$, which maps the input image X to high-dimensional feature representations.

Freezing a layer means preventing its weights from being updated during training. Mathematically, for each trainable parameter $\:{\theta\:}_{i}$ in the model, we set:

$$\:\nabla\:{\theta\:}_{i}L\left(\theta\:\right)=0\:\:\:\:\forall\:\:i\:\in\:\:\{1,\:2,\:.\:.\:.\:,\:L\}$$

where L(θ) is the loss function. This ensures:

- The pre-trained weights $\:{\theta\:}_{i}$ remain unchanged.
- The model acts purely as a fixed transformation: $\:X\to\:f\left(X\right)$

Next, a new Keras Model is created that maps:

$$\:f\left(X\right)=CNN\left[0:-4\right]\left(X\right)$$

This model uses the same input layer as the original model but stops at the fourth-to-last layer (after the classification layers have been removed). The result is a truncated CNN that outputs a tensor of shape:

$$\:f\left(X\right)\in\:{R}^{n\times\:h\times\:w\times\:d}$$

where:

- n is the number of samples,
- h, w are the spatial dimensions,
- d is the number of feature channels.

Each input image $\:X\left(i\right)\in\:{R}^{H\times\:W\times\:C}$ is passed through the truncated CNN, resulting in a feature map:

$$\:f\left({X}^{\left(i\right)}\right)={T}^{\left(i\right)}\in\:{R}^{h\times\:w\times\:d}$$

The output tensor contains local features (from convolutional filters) aggregated spatially. This transformation captures the semantic information learned during pre-training.

Each 3D feature map $\:{T}^{\left(i\right)}\in\:{R}^{h\times\:w\times\:d}$ is flattened into a 1D vector:

$$\:{v}^{\left(i\right)}=flatten\left({T}^{\left(i\right)}\right)\in\:{R}^{m}\:\:\:\:\:where\:m=h\times\:w\times\:d$$

This converts the full set into a 2D feature matrix:

$$\:{X}_{features}\in\:{R}^{n\times\:m}$$

Given input images $\:X\in\:{R}^{n\times\:H\times\:W\times\:C}$, the pipeline transforms them as follows:

$$\:X\underrightarrow{Truncated\:CNN}f\left(X\right)\in\:{R}^{n\times\:h\times\:w\times\:d}\underrightarrow{Flatten}{R}^{n\times\:m}$$

The final feature matrix can then be used directly for classification, clustering, or other downstream tasks.

### Machine learning classifier

#### Support vector machine classifier

When it comes to classification, few supervised learning algorithms are as effective as Support Vector Machine (SVM). Finding the optimal boundary (a hyperplane) that divides data points into multiple groups is the fundamental premise of support vector machines (SVM). A machine learning model called support vector machine (SVM) will not pick a border at random but will instead look for the one that maximizes the distance, or the margin, across it and the nearest points of data in every category^42^. Because they specify the location and direction of the ideal border, these nearest points—called support vectors—are crucial. Support vector machines (SVMs) may keep running with non-linearly separable data by utilizing kernel functions^42^. This occurs when the data is elevated to a higher-dimensional space, making the possibility of a dividing line more realistic. When dealing with high-dimensional data, support vector machines (SVMs) find widespread use in bioinformatics, picture classification, and text classification.

The first approach uses a pre-trained CNN as a full feature extractor with all CNN layers frozen. A Support Vector Machine (SVM) classifier is trained using the output feature vectors. Since the CNN layers are frozen, no part of the feature extraction network is updated during training. The SVM then learns a hyperplane (or multiple, in multi-class settings) that best separates the classes in this fixed high-dimensional space. The decision boundary is defined mathematically as:

$$\:\widehat{y}=sign({w}^{T}x+b)$$

where x is the feature vector, w is the weight vector, and b is the bias term.

The second approach involves removing all but the convolutional base from the CNN, which includes all of its classification layers (including fully connected layers, softmax, and global average pooling). In addition to serving as a general-purpose feature extractor, these frozen convolutional layers are not changed during training. The last convolutional layer produces a tensor with many dimensions that includes both local and geographic data. The SVM classifier receives this tensor after it has been flattened into a one-dimensional vector. Despite their increased dimensionality, these feature vectors can keep richer spatial characteristics than the first technique and may be more effective for some applications. Once again, the best hyperplane for class separation is determined by training the SVM on these vectors that have been flattened.

#### Decision tree classifier

For classification problems, a supervised learning technique known as a Decision Tree Classifier can be employed. To work, it uses input features to recursively divide the feature space into subsets, forming a tree-like structure with class labels at the leaf nodes and decisions at the inside nodes^43^. A decision tree’s main purpose is to partition the data such that distinct classes are as far apart as possible, with the objective of creating progressively more homogenous branches with regard to the dependent variable.

The technique utilizes impurity metrics like Gini impurity or entropy (information gain based) to find the optimal feature and threshold for data splitting at each node. The Gini impurity at a given node t may be determined by:

$$\:G\left(t\right)=1-\sum\:_{i=1}^{C}{p}_{i}^{2}$$

and for entropy, it is

$$\:H\left(t\right)=-\sum\:_{i=1}^{C}{p}_{i}{\mathrm{log}}_{2}\left({p}_{i}\right)$$

C is the number of classes, and $\:{p}_{i}$ is the fraction of samples belonging to class i at that node. By iteratively building branches, the method finds the feature that reduces impurity the most and continues doing so until either a maximum depth is reached, a minimum number of samples per node is reached, or the node becomes pure.

The first approach involves using a Decision Tree Classifier after flattening all of the retrieved features. This feature space is iteratively divided into smaller, more homogenous parts by the decision tree using these fixed feature vectors. By utilizing metrics such as Gini impurity or entropy, the model chooses a feature and a threshold at each internal node of the tree to partition the data in a manner that optimizes the decrease of impurity.

In this second strategy, feature vectors are often higher-dimensional and richer in spatial semantics than those obtained from the fully frozen CNN. Because of this, the input to the decision tree may contain more detailed information about local structures in the image (like object parts, edges, or texture variations).

The Decision Tree Classifier then takes these rich, fixed features and learns a sequence of if-else rules that split the feature space to classify samples. The training goal is the same: to maximize class separation using a series of feature-based splits.

At each node, the decision tree selects a feature xj and a threshold θ such that the data is split as:

$$\:Split:\:{x}_{j}<\theta\:\:\mathrm{o}\mathrm{r}\:{x}_{j}\ge\:\theta\:$$

The best split is chosen to maximize information gain or minimize impurity. The decision path for each input leads to a leaf node corresponding to a predicted class.

#### K-Nearest neighbors classifier

When it comes to classification and regression, the K-Nearest Neighbors (K-NN) algorithm is your go-to non-parametric option. For classification purposes, it finds the k feature points that are geographically nearest to a test sample and uses the most frequent class among those k to predict the label for the test sample^44^. The K-NN model is unique in that it uses the stored training data and distances between points as its only inputs, rather than explicitly establishing a decision boundary.

Mathematically, for a test feature vector x, the predicted label $\:\widehat{y}$ is:

$$\:\widehat{y}=mode(\left\{{y}_{i}\right|{x}_{i}\in\:{N}_{k}(x\left)\right\})$$

where:

- $\:{N}_{k}\left(x\right)$ is the set of the k-nearest neighbours to x,
- $\:{y}_{i}\:$are the labels of those neighbours.

In the first approach, a pre-trained CNN is used to extract deep feature vectors from images. The final output is typically a dense feature vector stored as part of the training dataset. During prediction, a test image is passed through the same CNN, producing a new feature vector. The Euclidean distance (or other metrics like cosine similarity) between the test vector and all training vectors is computed to find the k closest matches:

$$\:distance\:\left(x,\:{x}_{i}\right)={‖x-{x}_{i}‖}_{2}$$

The class label is then determined by majority vote among the k nearest training vectors.

In this second approach, the classification layers of the CNN (global average pooling, fully connected layers, dropout, softmax) are removed, and only the convolutional base is retained. The key difference here is the richer feature representation. The output from the final convolutional layer is typically a 3D tensor,

$$\:T\in\:{R}^{h\times\:w\times\:d}$$

which is flattened into a 1D vector:

$$\:x=flatten\left(T\right)\in\:{R}^{m},\:\mathrm{w}\mathrm{h}\mathrm{e}\mathrm{r}\mathrm{e}\:m=h\bullet\:w\bullet\:d$$

These higher-dimensional vectors retain more spatial information and can be more discriminative. Once flattened, the K-NN classifier proceeds identically as in the first approach: compute distances, find the k-nearest, and assign the majority class.

#### Random forest classifier

Classification and regression hitches are well-suited to the ensemble learning approach known as a Random Forest. During training, it builds a network of decision trees, or a “forest”, and then uses the mean of those trees’ predictions to determine the output class^45^. It improves accuracy and avoids overfitting by averaging the output of numerous decision trees learned on different data and subsets of features.

This initial approach involves feeding an input picture into a convolutional neural network (CNN). The CNN produces a compact feature vector, which is then fed into a Random Forest classifier. The Random Forest classifier learns to differentiate between classes by building several decision trees. Through the use of bagging and feature sub-sampling, each tree is trained using a randomly selected portion of the training data and features. Using majority voting, we compile all of the trees’ predictions and use them to make a final class prediction for a test picture.

Mathematically, for a feature vector $\:x\in\:{R}^{d}$, and trees $\:{T}_{1},{T}_{2},{T}_{3},{T}_{4},{T}_{5},\dots\:\dots\:..,{T}_{M}$, the prediction is:

$$\:\widehat{y}=mode({T}_{1}\left(x\right),{T}_{2}\left(x\right),{T}_{3}\left(x\right),\dots\:\dots\:\dots\:.,{T}_{M}(x\left)\right)$$

where.

- M is the number of trees in the forest.
- $\:{T}_{i}\left(x\right)\:$is the predicted class label from the i-th decision tree.

In the second approach, the output from the final convolutional layer is typically a 3D tensor representing spatial and channel-wise features. This tensor is flattened into a 1D feature vector (often higher-dimensional than in the first approach due to preserved spatial features):

$$\:x=flatten\left(T\right),\:\mathrm{w}\mathrm{h}\mathrm{e}\mathrm{r}\mathrm{e}\:T\:\in\:\:{R}^{h\times\:w\times\:d},\:x\in\:{R}^{m},\:m=h\bullet\:w\bullet\:d$$

These rich, high-dimensional vectors are then used as inputs to train the Random Forest classifier. Because these features preserve more local information (such as edges, textures, and spatial positions), the Random Forest can potentially perform better on tasks where spatial patterns are crucial.

#### Logistic regression classifier

Logistic Regression, a model that uses linear regression, is utilized for binary and multi-class classification tasks. It employs the logistic (sigmoid) or softmax (multi-class) functions to articulate the probability that an input vector is associated with a certain class^46^. The logistic regression function is employed for the classification of binary data.

Mathematically, for binary classification:

$$\:P\left(y=1|x\right)=\sigma\:\left({w}^{T}x+b\right)=\frac{1}{1+{e}^{-({w}^{T}x+b)}}$$

For multi-class classification with C classes:

$$\:P\left(y=c|x\right)=\frac{{e}^{{w}_{c}^{T}\:x\:+\:{b}_{c}}}{{\sum\:}_{j=1}^{C}{w}_{j}^{T}\:x\:+\:{b}_{j}}\:\mathrm{f}\mathrm{o}\mathrm{r}\:c=1,\dots\:\dots\:\dots\:.,C$$

where:

- $\:x\in\:{R}^{d}$ is the feature vector,
- $\:{w}_{c},{b}_{c}$ are the weight vector and bias term for class c,
- $\:\sigma\:$ is the sigmoid function.

The output of the model is the class with the highest predicted probability.

In the first approach, feature vectors are passed to the Logistic Regression classifier, which learns to linearly separate the classes in this feature space. Despite being a linear model, Logistic Regression often performs well on such CNN features because the convolutional network already projects images into a highly informative representation space.

Prediction is made using the softmax (or sigmoid for binary) function, and training is done by minimizing the cross-entropy loss.

The cross-entropy loss for multi-class classification using logistic regression is given by:

$$\:L=-\sum\:_{i=1}^{N}\sum\:_{c=1}^{C}{y}_{i,c}\mathrm{log}P(y=c|{x}_{i})$$

where:

- $\:{y}_{i,c}\in\:\left\{\mathrm{0,1}\right\}$ is the ground truth indicator for sample i belonging to class c ,
- N is the total number of samples,
- $\:P\left(y=c|{x}_{i}\right)$ is the predicted probability of class c given input $\:{x}_{i}$.

In the second approach, feature vectors are then passed to the Logistic Regression classifier. The rest of the classification process is the same: the classifier tries to learn linear decision boundaries in this high-dimensional space to predict class probabilities via softmax.

### Overall framework walkthrough

**Fig. 6:**
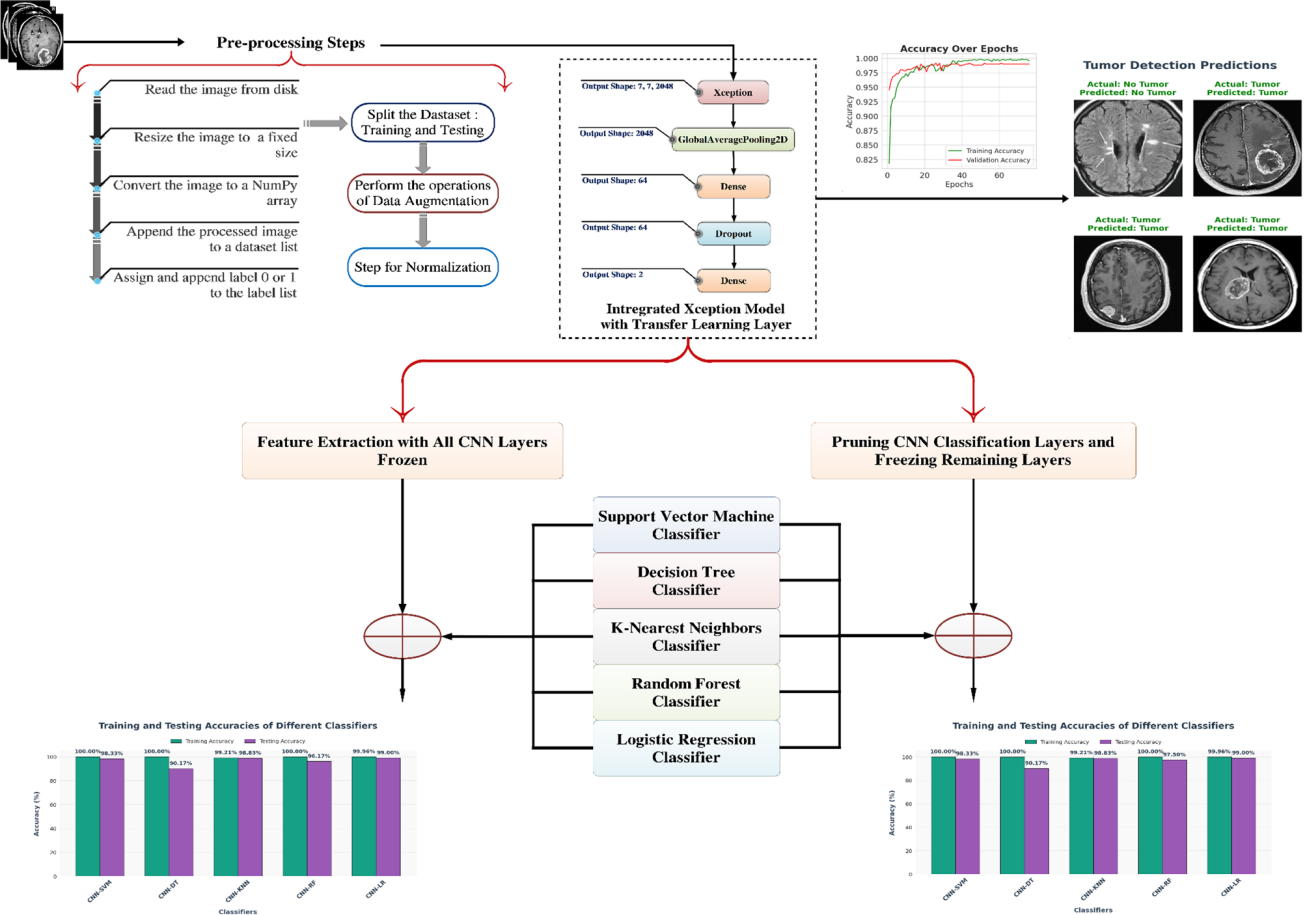
Overall framework walkthrough.

The proposed approach [Figure 6] combines deep neural network extraction of features with traditional machine learning classifiers to efficiently classify brain tumors using MRI data. The pipeline starts with a preliminary processing phase, during which brain MRI pictures are methodically retrieved from disk. Every picture is downsized to a standardized resolution to maintain uniformity in input dimensions for the deep neural network. These resized images are then transformed into NumPy arrays, facilitating efficient computational handling. Subsequently, each preprocessed image is appended to a dataset list, while the corresponding binary class label—indicating tumor presence (1) or absence (0)—is appended to a label list. This structured dataset serves as the foundation for feature learning and classification. Following preprocessing, images are passed into the Xception model, a convolutional neural network known for its use of depthwise separable convolutions, enabling efficient and scalable deep feature extraction. Two distinct strategies for feature extraction are employed in this framework:

- In the first approach, the entire Xception model is retained in its pre-trained form, with all weights frozen. The model acts solely as a static feature extractor, and no backpropagation updates are performed on its layers. Deep feature vectors are obtained from the final output of the network, typically after the GlobalAveragePooling2D layer, resulting in compact yet semantically rich representations of the input images. These feature vectors, generally of fixed lower dimensionality are then used as input to a suite of conventional classifiers, including Support Vector Machine (SVM), Decision Tree (DT), K-Nearest Neighbors (K-NN), Random Forest (RF), and Logistic Regression (LR). Each classifier is trained to learn the optimal decision boundary that separates the tumor and non-tumor classes in the feature space.
- The second strategy introduces a key variation by removing the classification layers of the Xception model, including the global pooling, dense, dropout, and softmax layers. Only the convolutional base is retained, and these layers are frozen to maintain the integrity of learned spatial features. The output from the last convolutional block is a 3D tensor of dimensions.

$$\:T\in\:{R}^{h\times\:w\times\:d}$$

capturing fine-grained spatial and hierarchical feature information. This tensor is subsequently flattened into a 1D vector, where the final feature dimension is computed as.

$$\:m=h\bullet\:w\bullet\:d$$

These high-dimensional vectors, while more computationally intensive, retain greater spatial and structural information compared to global-pooled features. They are then supplied to the same ensemble of machine learning classifiers used in the first strategy.

This hybrid architecture effectively combines the representation power of pre-trained CNNs with the interpretability and flexibility of classical classifiers. The dual-strategy approach offers two complementary paradigms for feature extraction: one favoring computational efficiency (global pooled vectors), and the other emphasizing spatial richness (flattened convolutional outputs). Such a framework proves particularly beneficial in medical imaging domains, where diagnostic accuracy and model reliability are paramount. Table 5 showing the details of hyperparameter.

**Table 5:** Comprehensive hyperparameter and experimental settings for the xcepfusion framework.

Category	Parameter	Description	Value / Setting
CNN feature extractor (Xception)	Base Model	Pre-trained model used for transfer learning	Xception (ImageNet weights)
	Input Image Size	Size of MRI images used for feature extraction	224 × 224 × 3
	Feature Extraction Strategy	Two strategies used	(1) All CNN layers frozen, (2) Pruned classification layers
	Optimizer	Optimization algorithm for fine-tuning	Adam
	Learning Rate	Step size for parameter updates	0.0001
	Batch Size	Samples processed before model weight update	32
	Epochs	Number of complete training iterations	70
	Loss Function	Objective function minimized during training	Binary Cross-Entropy
	Activation Function	Non-linear activation functions	ReLU (hidden layers), Sigmoid (output layer)
	Dropout Rate	Regularization rate to prevent overfitting	0.5
	Weight Initialization	Initialization technique	He Normal
	Data Augmentation	Techniques to increase generalization	Rotation, flipping, zooming, and contrast adjustment
Traditional ML classifiers	Support Vector Machine (SVM)	Kernel and regularization settings	Kernel = RBF, C = 1.0, Gamma = ‘scale’
	K-Nearest Neighbors (KNN)	Number of neighbors used	k = 5, Distance = Euclidean
	Random Forest (RF)	Ensemble parameters	n_estimators = 100, max_depth = 10
	Decision Tree (DT)	Splitting criterion and max depth	Criterion = ‘gini’, max_depth = 10
	Logistic Regression (LR)	Regularization method and solver	Penalty = L2, Solver = ‘lbfgs’, Max_iter = 1000
Training environment	Hardware	Computational setup used for experiments	Google Colab (High-RAM) with NVIDIA A100 GPU (40GB VRAM)
	Software	Frameworks and libraries	TensorFlow 2.13, scikit-learn 1.3, Keras, Python 3.10

## Measurement parameter

### Accuracy: the overall verdict

Accuracy as the final score of the model — it’s the fraction of correct decisions out of all decisions made. In other words, it answers the question:

“Out of everything the model tried to classify, how often was it right?”

Accuracy is defined as:

$$\:Accuracy=\frac{TP+TN}{TP+FP+TN+FN}$$

where:

- TP (True Positives): Correctly predicted tumor cases.
- TN (True Negatives): Correctly predicted non-tumor cases.
- FP (False Positives): Non-tumor cases incorrectly predicted as tumor.
- FN (False Negatives): Tumor cases missed by the model.

### Loss: the cost of being wrong

Loss is the model’s internal disappointment. It quantifies how far the predictions are from the actual labels, forming the core signal that guides model learning during training.

One popular function of loss for classification with multiple classes applications is categorical cross-entropy. It is a measure of how different the actual class distribution is from the projected probability distribution. As the estimated likelihood moves more away from the real label, the loss grows.

Let:

- N be the number of samples,
- C be the number of classes,
- $\:{y}_{i,c}\in\:\{0,\:1\}$be the ground truth label for class c of sample i,
- $\:{\widehat{y}}_{i,c}\in\:[0,\:1]\:$be the predicted probability that sample i belongs to class c.

The Categorical Cross-Entropy loss is given by:

$$\:L=-\sum\:_{i=1}^{N}\sum\:_{c=1}^{C}{y}_{i,c}\mathrm{log}\left({\widehat{y}}_{i,c}\right)$$

This formulation ensures that the loss is minimized when the predicted probability for the correct class approaches 1, making it a powerful objective for training classifiers in deep learning.

### Precision: how reliable are positive predictions?

Precision tells us: “When the model predicts positive, how often is it correct?” This metric is particularly important in scenarios where false positives carry a significant cost, such as in medical diagnostics or fraud detection.

Precision is defined as:

$$\:Precision=\frac{TP}{TP+FP}$$

### Recall: the sensitivity radar

Recall is like a safety net that ensures the model doesn’t miss anything important especially critical in high-stakes fields like medicine. It answers the question:

Out of all the actual positive cases, how many did the model successfully identify?

Recall is defined as:

$$\:Recall=\frac{TP}{TP+FN}$$

In other words, recall measures how sensitive the model is in detecting positive cases. For instance, in tumor detection, it is preferable to raise a false alarm (which can be verified by further testing) than to miss a real tumor. A model with high recall helps minimize such dangerous false negatives, making it a crucial metric in medical diagnostics and other critical applications.

### F1-score — balance between precision and recall

The F1-Score represents the harmonic mean of accuracy and recall. It offers a singular metric that reconciles both issues, particularly advantageous for unbalanced datasets.

$$\:F1-Score=\frac{2\bullet\:Precision\bullet\:Recall}{Precision+Recall}$$

### ROC curve— threshold-independent performance

The Receiver Operating Characteristic (ROC) curve illustrates the True Positive Rate (TPR) in relation to the False Positive Rate (FPR) across different categorization thresholds.

Mathematically, these are defined as:

$$\:TPR=\frac{TP}{TP+FN},\:FPR=\frac{FP}{FP+TN}$$

This curve illustrates the trade-off between sensitivity (recall or true positive rate) and the false positive rate (1 minus specificity) across several threshold settings. A curve that approaches the top-left corner signifies superior performance.

## Results

**Fig. 7:**
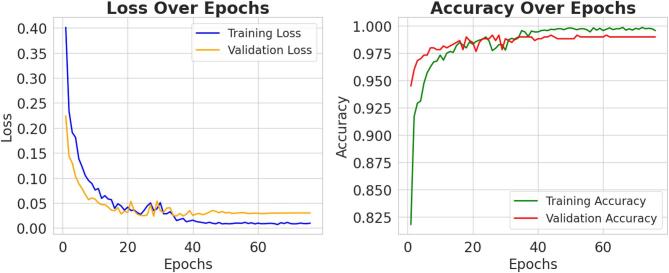
Loss and accuracy graph over epochs.

Using loss and accuracy measures on training and validation datasets, Fig. 7; Table 6 show the training dynamics of a machine learning model across 70 epochs. With each passing epoch, the training loss is less, which means the error function is becoming minimized. At the same time, the validation loss decreases in a comparable pattern and settles at a low value, indicating that the model retains its capacity to generalize without substantial overfitting.

Training accuracy improves over time, eventually reaching a near-optimal level approaching convergence, when considering performance. Maintaining a high and consistent number, validation accuracy closely resembles training accuracy, suggesting strong performance on unseen data. High generalizability and low variance are results of an optimized model with well-regularized parameters, as shown by training and validation metrics that are in agreement with one another.

**Table 6:** Loss and accuracy for training and testing for integrating Xception with transfer learning Layer.

	Accuracy	Loss
Training	0.9950	0.0331
Testing	0.9900	0.0259

**Fig. 8:**
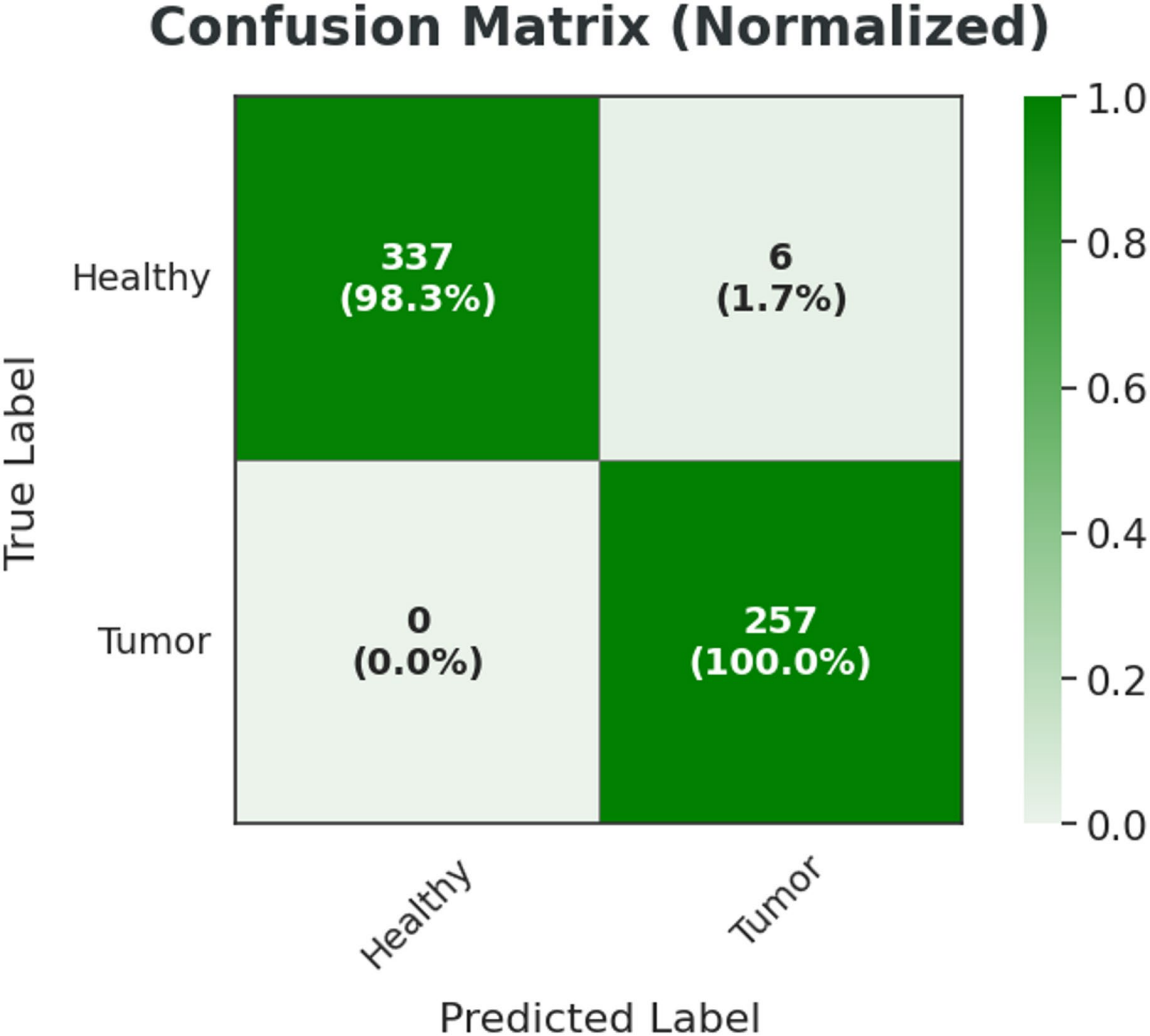
Confusion matrix (Normalized).

Using the Xception architecture linked with a transfer learning layer, this confusion matrix Fig. 8 offers an evaluation of a binary classification model meant to separate healthy from tumour samples. The model reportedly attained exceptional categorisation accuracy. Medical diagnosis depends on the elimination of false negatives as it reduces the possibility of missing a crucial diagnosis. The very remarkable accuracy of the model is further improved by the low frequency of false positives. Particularly in high-stakes domains like medical image analysis, the results reveal that integrating Xception with transfer learning lets the model collect strong and unique characteristics, hence producing outstanding classification performance and great generalisation.

**Fig. 9:**
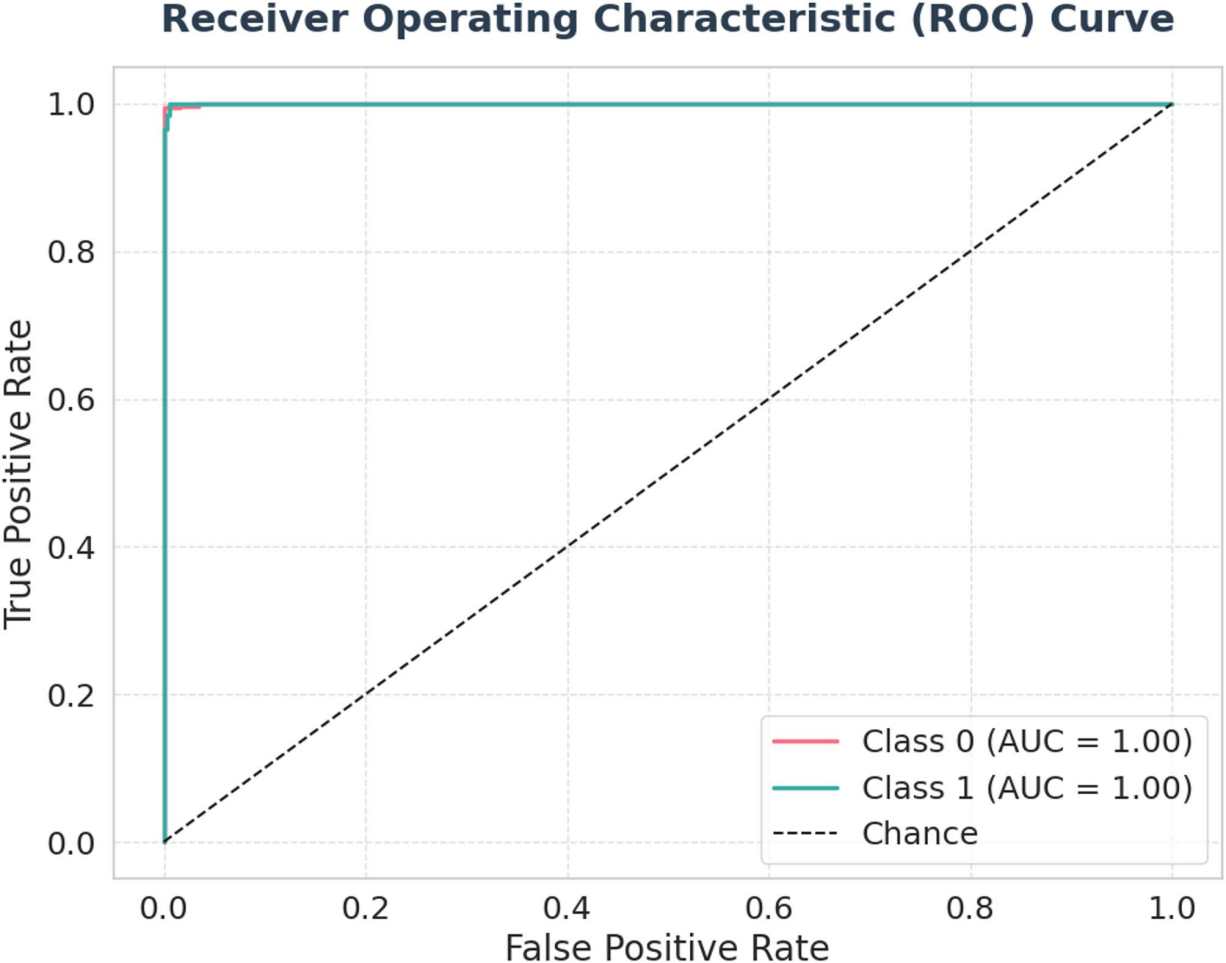
Graph plot - RoC curve.

Using the Xception model coupled with a transfer learning layer, this ROC (Receiver Operating Characteristic) curve [Figure 9] shows how well a binary classifier performs. Under various threshold settings, the figure evaluates whether the model can distinguish between two categories—probably “healthy” and “tumour”. Both Class 0 and Class 1 attain an Area Under the Curve (AUC) of 1.00, so the curve shows remarkable classification performance. With a value of 1.0 representing ideal performance, the AUC is a comprehensive statistic that captures the ability of the model to differentiate across classes.

With practically no false positive rate and a value of 1.0 almost instantaneous, the sensitivity shows that the model can effectively detect almost all positive cases while avoiding labelling negative instances mistakes. By using transfer learning with the Xception architecture, the model may use pre-trained feature representations, hence enhancing its ability to detect complex patterns in medical imaging data and producing remarkable discriminative performance.

**Fig. 10:**
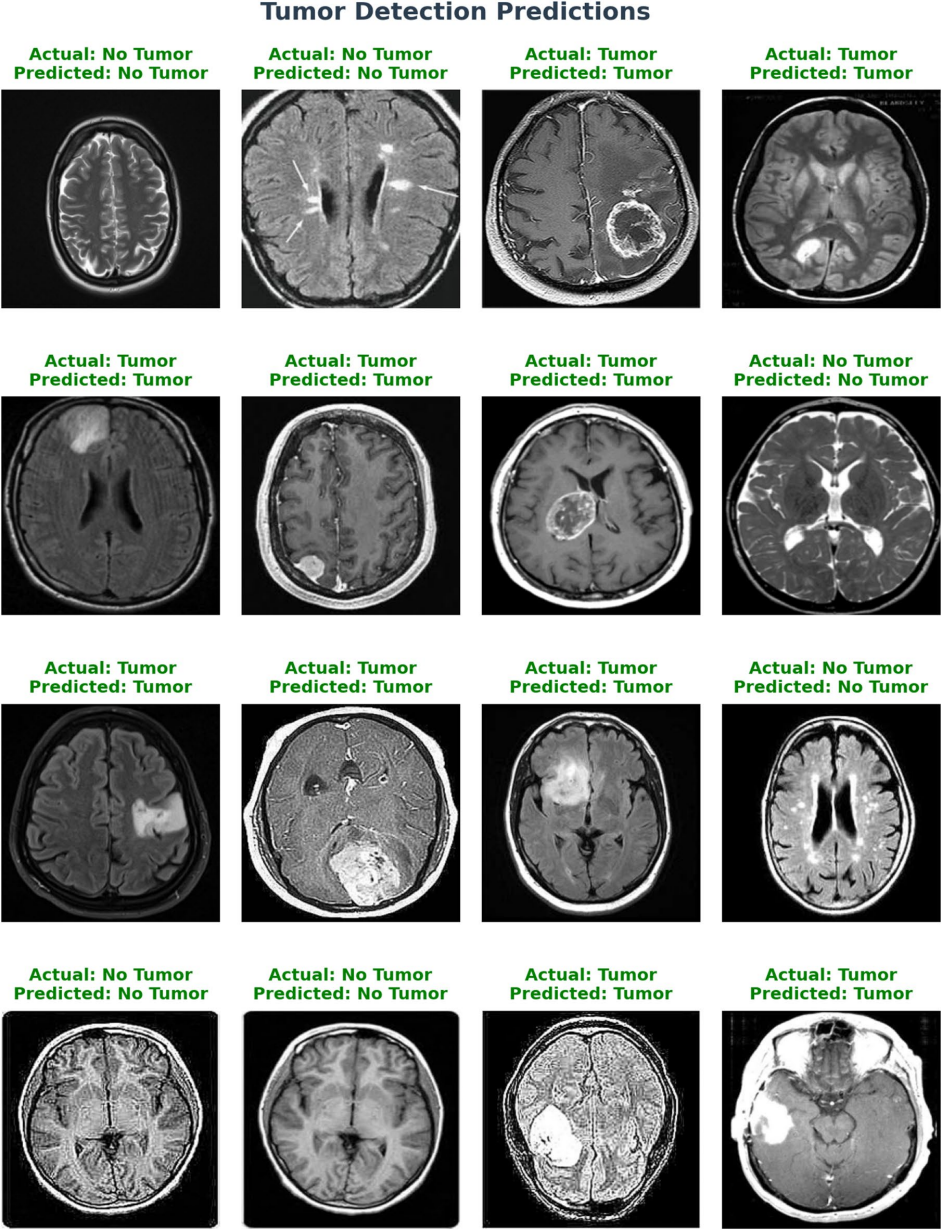
Tumor detection prediction.

Using Xception architecture in conjunction with a transfer learning layer, a classification model shows the cancer detection performance in Fig. 10—a collection of qualitative data. Grid displays from MRI brain images, each annotated to illustrate the ground truth diagnosis with the model’s forecast. Featuring a transfer learning layer and the Xception architecture, Model 4’s classification report shows the encouraging efficiency of this method for binary medical picture classification problems. Key measures of a classification model’s performance include recall, precision, and F1-score [Table 7]. While avoiding false positives, accuracy evaluates the model’s capacity to accurately detect pertinent events. Recall finds all relevant events, therefore helping to minimise false negatives in the model. Combining accuracy and recall, the F1-score provides a harmonic mean of these two measures.

**Table 7:** Classification report for integrating Xception with transfer learning Layer.

	Precision	Recall	F1-Score
**0**	1.0000	0.9900	1.0000
**1**	0.9900	1.0000	0.9900
**Macro Avg**	0.9900	1.0000	0.9900
**Weighted Avg**	1.0000	1.0000	1.0000

**Fig. 11:**
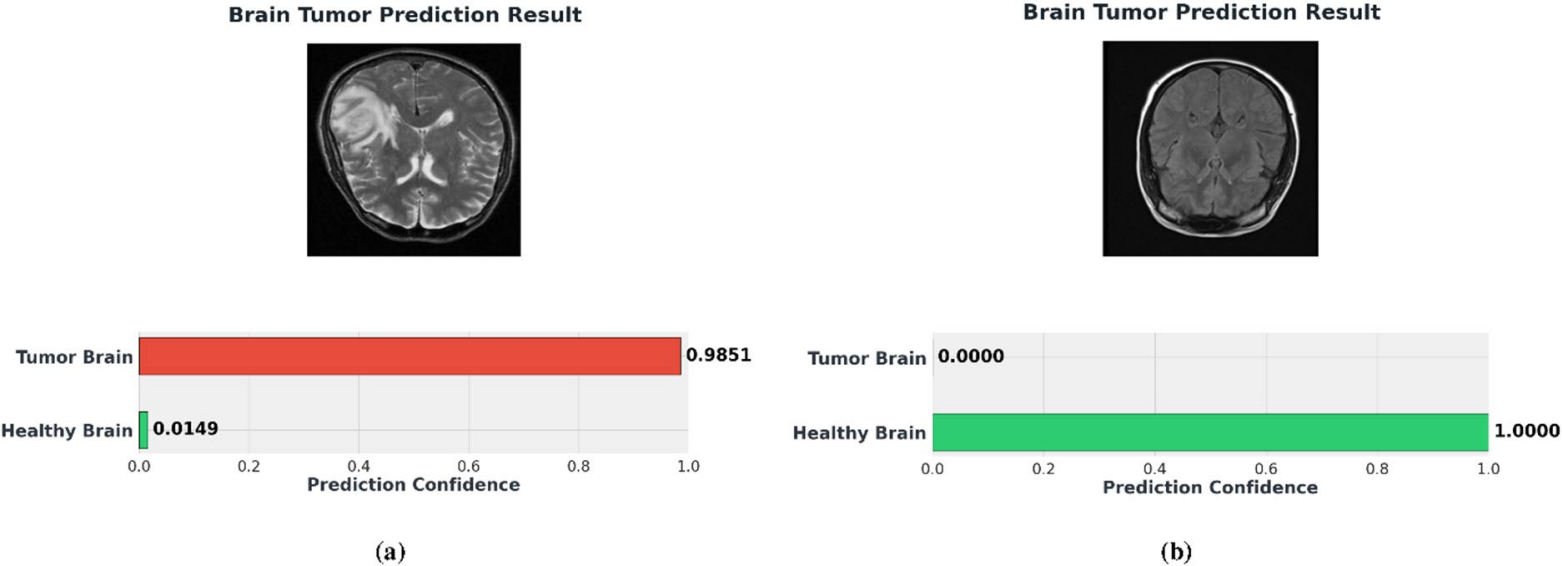
Brain tumor prediction confidence result.

The prediction results [Figure 11] reflect the model’s capability to assign high confidence to the correct class, showcasing the effectiveness of deep feature extraction achieved through Xception’s convolutional layers and the refinement brought by transfer learning. In the first case, the model strongly predicts the presence of a tumor, indicating high confidence for the tumor brain class and very low confidence for the healthy brain class. In contrast, the second case illustrates a scenario where the model confidently identifies the image as representing a healthy brain, assigning full confidence to that class while completely excluding the possibility of a tumor.

**Table 8:** Training and testing accuracies of different Classifiers - Feature extraction with all CNN layers Frozen.

Classifier	Training Accuracy	Testing Accuracy
CNN-SVM	1.0000	0.9833
CNN-DT	1.0000	0.9017
CNN-KNN	0.9904	0.9850
CNN-RF	1.0000	0.9750
CNN-LR	0.9996	0.9900

**Fig. 12:**
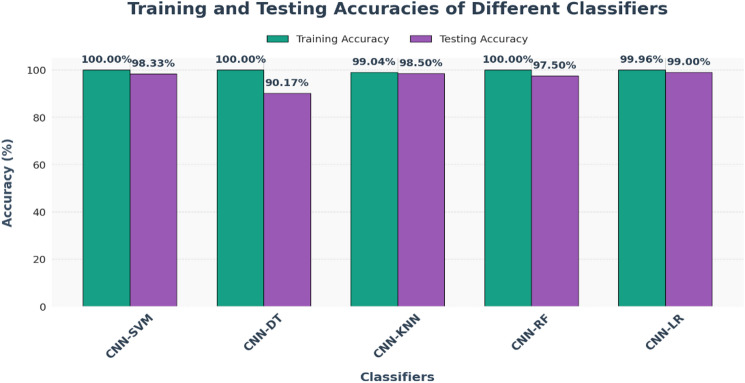
Training and testing accuracies of different classifiers - Feature extraction with all CNN layers frozen.

From the Fig. 12; Table 8, it is evident that all models achieved near-perfect training accuracy, indicating they were able to learn the patterns in the training set effectively. However, the testing accuracies vary, revealing the generalization ability of each model. While some models like CNN-SVM and CNN-KNN maintain high testing accuracy with minimal drop from training, others like CNN-DT show a more significant gap, suggesting possible overfitting.

**Fig. 13:**
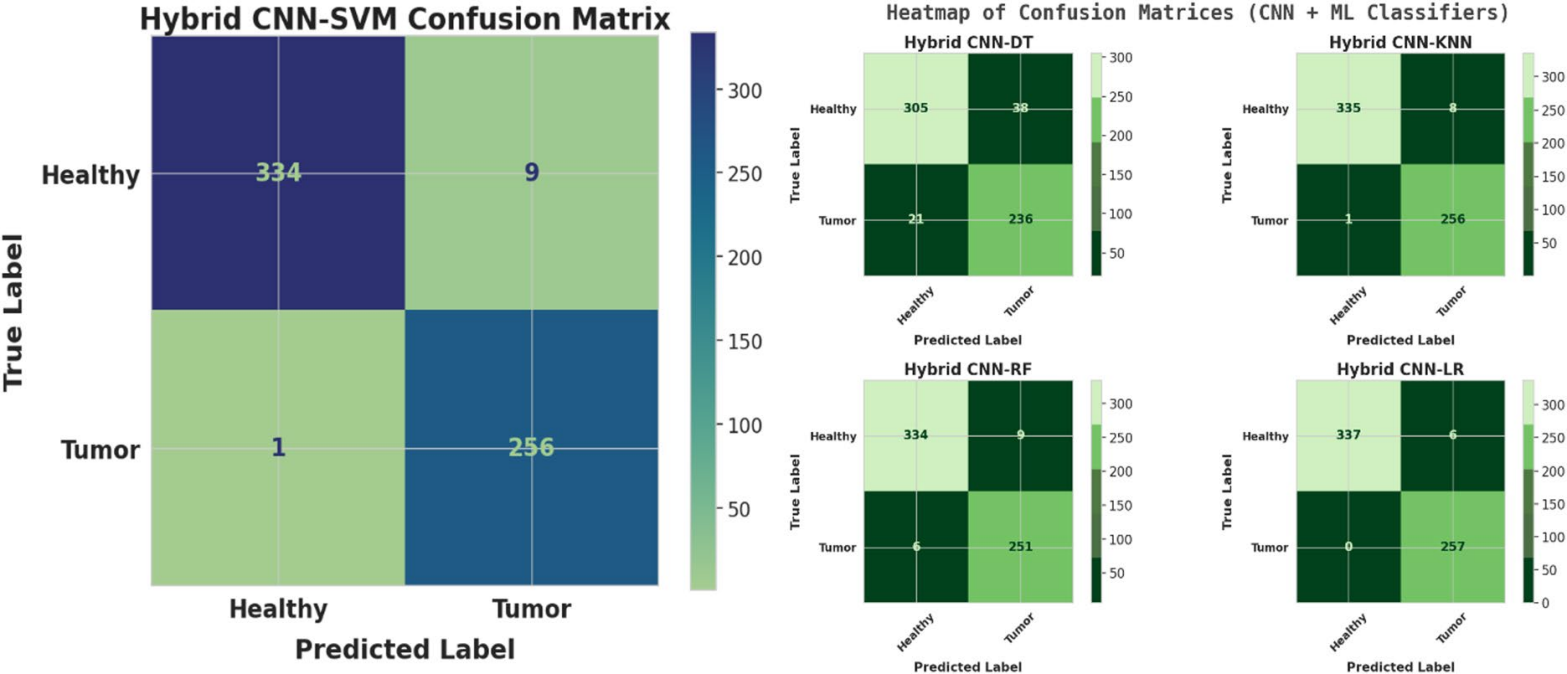
Confusion matrix - Feature extraction with all CNN layers frozen.

This Fig. 13 demonstrates the effectiveness of using a CNN as a fixed feature extractor, where all layers of the CNN are frozen and not updated during training. In this setup, the CNN serves solely to extract deep features from the input data, which are then passed to traditional machine learning classifiers for final classification. On the left side, the confusion matrix displays the performance of the hybrid CNN-SVM model. Despite the CNN being entirely frozen, the SVM classifier performs strongly, indicating that the features captured by the pretrained CNN are highly informative and suitable for distinguishing between healthy and tumor categories. The right side of the figure includes confusion matrices for additional hybrid models that use different classifiers: Decision Tree, K-Nearest Neighbors, Random Forest, and Logistic Regression. Among these, Logistic Regression stands out with the most accurate classification performance, followed closely by K-Nearest Neighbors and Random Forest. The Decision Tree model shows comparatively lower accuracy, suggesting it may be less effective in utilizing the high-dimensional features extracted by the frozen CNN.

**Fig. 14:**
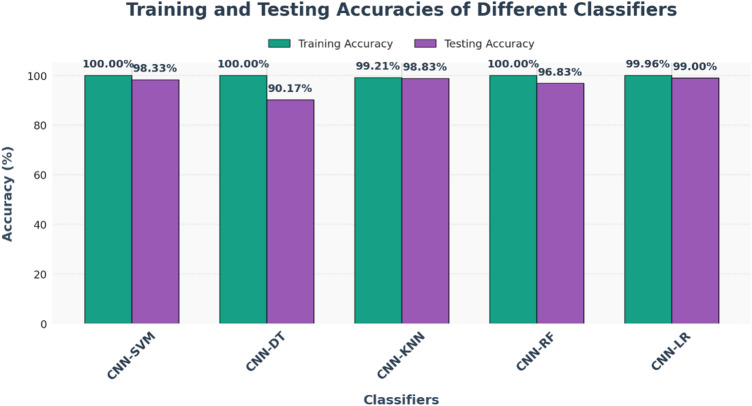
Training and Testing accuracies of different classifiers - Pruning CNN classification layers and freezing remaining layers.

**Fig. 15:**
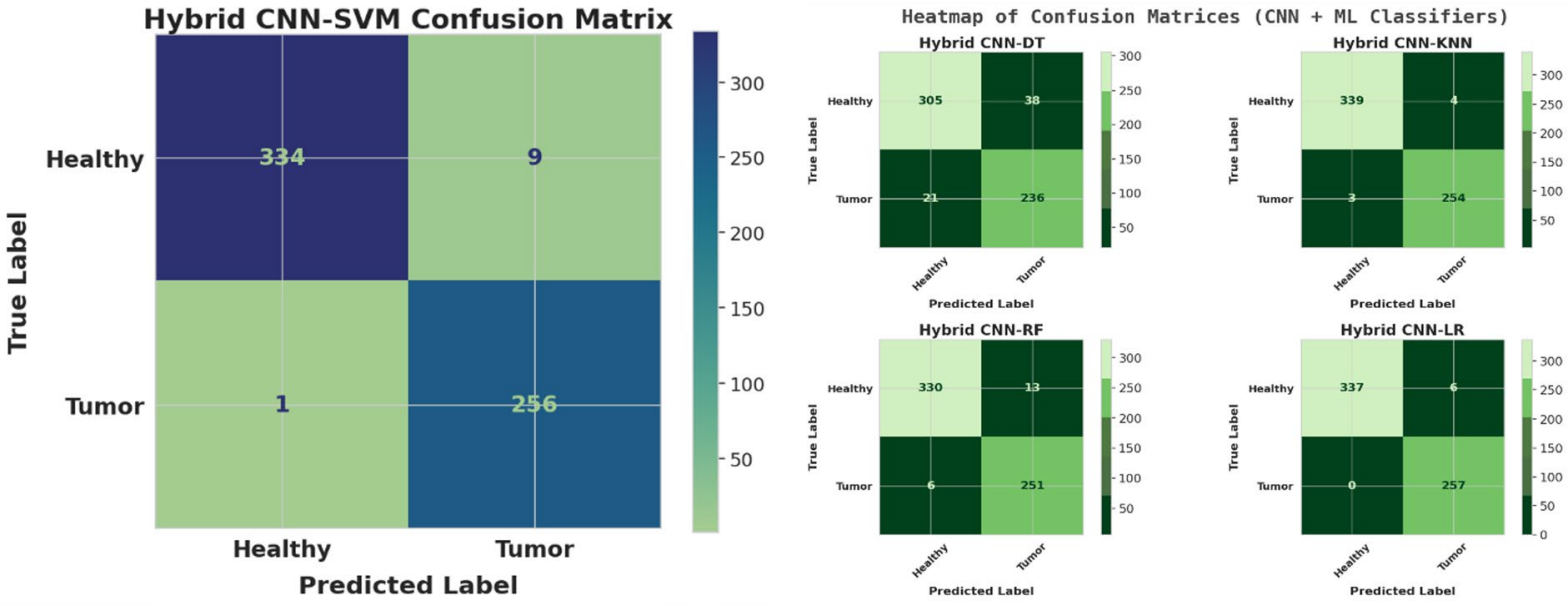
Confusion Matrix - Pruning CNN classification layers and freezing remaining layers.

Each classifier in this set uses a combination of Convolutional Neural Networks (CNN) and one or more machine learning models, such as Support Vector Machine (SVM), Decision Tree (DT), K-Nearest Neighbors (KNN), Random Forest (RF), or Logistic Regression (LR). Table 9; Fig. 14 show the results of these comparisons. An improved fit between the training data and the model’s output is indicated by a greater training accuracy. However, the capacity of the model to generalize to previously unknown data is assessed by accuracy testing. Figure 15 shows the confusion matrix for each classifier.

The results show that classifiers, such as CNN-SVM, CNN-RF, and CNN-LR, achieve perfect training accuracy, meaning they fit the training data perfectly. However, their testing accuracy slightly decreases, suggesting that while these models perform excellently on the training set, they face some challenges in generalizing to the test set. The CNN-DT classifier also achieves perfect training accuracy, but its testing accuracy drops significantly, indicating overfitting, where the model performs well on the training data but struggles with unseen data. In contrast, the CNN-KNN classifier demonstrates a smaller gap between its training accuracy and testing accuracy, indicating better generalization and less overfitting.

**Table 9:** Training and testing accuracies of different Classifiers - Pruning CNN classification layers and freezing remaining layers.

Classifier	Training Accuracy	Testing Accuracy
CNN-SVM	1.0000	0.9833
CNN-DT	1.0000	0.9017
CNN-KNN	0.9921	0.9883
CNN-RF	1.0000	0.9683
CNN-LR	0.9996	0.9900

## Discussion

The efficiency comparison in Table 10 demonstrates that the pruned Xception configuration achieves substantial computational savings, supporting its potential integration into clinical decision-support systems where inference speed is critical.

**Table 10:** Configuration.

Configuration	Trainable parameters	Model size (MB)	Training time (per epoch)	Inference time (per image)
Full layer freezing	0 (All layers frozen)	85	~ 45 s	0.021 s
Pruned classification layers	≈ 2.5 M	62	~ 38 s	0.018 s

Table 11 provides a comprehensive comparison of recent methodologies applied to brain tumor classification using MRI datasets.

**Table 11:** Comparison with other State-of-the-Art Methodologies.

References	Methodology	Dataset	Classification scope	Accuracy
Agarwal et al. (2024)^20^	Inception V3	Figshare “Brain Tumor” MRI dataset	Multi-class (glioma / meningioma / pituitary / healthy)	0.9889
Agarwal et al. (2024)^20^	Multi-Path CNN	Figshare “Brain Tumor” MRI dataset	Multi-class (glioma / meningioma / pituitary / healthy)	0.9600
Malakouti et al. (2024)^36^	LightGBM + Transfer Learning	MRI tumor classification dataset	Binary (tumor vs. non-tumor)	0.9570
Pande et al. (2024)^47^	INDEMNIFIER	Diverse MRIs (various sources)	Binary (tumor vs. non-tumor)	0.9730
Yoon et al. (2025)^48^	PDCNN	MRI (hybrid ensemble)	Multi-class (tumor-type & healthy)	0.9485
Malebary et al. (2024)^49^	Multi-Layer Hybrid U-Net + CNN	MRI segmentation/classification dataset	Multi-class + segmentation	0.9700
Kaur et al. (2025)^50^	Transfer-Learning Optimized ResNet152	MRI brain tumor classification dataset	Binary (tumor vs. non-tumor)	0.9853
Nizamani et al. (2023)^51^	FE1-HU-NET	MRI tumor segmentation + classification dataset	Multi-class + segmentation	0.9870
Nizamani et al. (2023)^51^	FE2-HU-NET	MRI tumor segmentation + classification dataset	Multi-class + segmentation	0.9860
Nizamani et al. (2023)^51^	FE3-HU-NET	MRI tumor segmentation + classification dataset	Multi-class + segmentation	0.9890
Nizamani et al. (2023)^51^	FE4-HU-NET	MRI tumor segmentation + classification dataset	Multi-class + segmentation	0.9760
Shoaib et al. (2024)^52^	DenseNet201 + PCA + SVM	MRI tumor classification dataset	Binary (tumor vs. non-tumor)	0.9800
Shoaib et al. (2024)^52^	DenseNet201 + PCA + MLP	MRI tumor classification dataset	Binary (tumor vs. non-tumor)	0.9800
Tiwari et al. (2024)^53^	ANFIS-F-DBN	MRI brain tumor classification dataset	Binary (tumor vs. non-tumor)	0.9000
Khushi et al. (2023)^54^	AlexNet architecture with SGD optimiser	BR35H: Brain–Tumor–Detection 2020 (Kaggle)	Binary (tumor vs. non-tumor)	0.9879
XcepFusion (All CNN layers frozen)	CNN-SVM	BR35H: Brain–Tumor–Detection 2020 (Kaggle)	Binary (tumor vs. non-tumor)	0.9833
	CNN-DT	BR35H: Brain–Tumor–Detection 2020	Binary (tumor vs. non-tumor)	0.9017
	CNN-KNN	BR35H: Brain–Tumor–Detection 2020	Binary (tumor vs. non-tumor)	0.9850
	CNN-RF	BR35H: Brain–Tumor–Detection 2020	Binary (tumor vs. non-tumor)	0.9750
	CNN-LR	BR35H: Brain–Tumor–Detection 2020	Binary (tumor vs. non-tumor)	0.9900
XcepFusion (Pruned CNN layers)	CNN-SVM	BR35H: Brain–Tumor–Detection 2020	Binary (tumor vs. non-tumor)	0.9833
	CNN-DT	BR35H: Brain–Tumor–Detection 2020	Binary (tumor vs. non-tumor)	0.9017
	CNN-KNN	BR35H: Brain–Tumor–Detection 2020	Binary (tumor vs. non-tumor)	0.9883
	CNN-RF	BR35H: Brain–Tumor–Detection 2020	Binary (tumor vs. non-tumor)	0.9683
	CNN-LR	BR35H: Brain–Tumor–Detection 2020	Binary (tumor vs. non-tumor)	0.9900

The dataset names have been corrected to the most precisely identified publicly available dataset for each work.Classification scope is clearly specified (binary vs. multi-class, segmentation or classification).

Combining the Xception model with transfer learning layers produced remarkable performance with a peak accuracy of.9900 using linear regression (CNN-LR). This was similar across two setups: one with feature extraction using all CNN layers frozen and the other with pruned classification layers while maintaining the remaining layers frozen, hence stressing the power of the Xception architecture in transfer learning techniques. In a same line, Nizamani et al.^48^ presented the Feature-Enhanced Hybrid U-Net (FE3-HU-NET), which showed the success of including sophisticated feature fusion approaches into hybrid deep learning models with an outstanding accuracy of.9890. By contrast, Agarwal et al.^17^ used the Inception V3 model, which achieved an accuracy of.9889, higher than their Multi-Path CNN arrangement, which recorded 0.9600. Investigated by Shoaib et al.^49^, the DenseNet 201 model consistently attained an accuracy of 0.9800 when combined with principal component analysis (PCA) and machine learning classifiers like SVM and MLP. This highlights how effectively methods for lowering feature dimensionality when paired with deep features work. Furthermore, the improved ResNet152 model created by Kaur et al.^46^ shown remarkable classification performance with an accuracy of.9853, therefore underlining the need of fine-tuning models in transfer learning environments. Combining light GBM with transfer learning produced an amazing accuracy of 0.9570^47^. By means of a much lower accuracy of.9000, the ANFIS-F-DBN model^50^ indicated that conventional neuro-fuzzy systems may have restrictions when compared to deep neural networks. Furthermore noted to have a significant effect on outcomes were variations in classifier choices, particularly in models based on Xception. For example, CNN combined with decision trees (CNN-DT) showed a performance level (about.9017), however using KNN and SVM produced noticeably higher accuracies (reaching up to.9883). The results show that adding advanced deep learning models—especially when combined with transfer learning and careful classifier selection—particularly helps to greatly increase the precision and dependability of brain tumour classification systems.While the proposed hybrid approach of leveraging Convolutional Neural Networks (CNNs) for feature extraction and traditional machine learning (ML) classifiers for brain tumor detection offers promising results, several limitations must be considered:

- Data Dependency and Imbalance: The availability of big, high-quality labelled datasets greatly determines the performance of CNNs and ML classifiers. Particularly in the field of brain tumour identification, these sometimes-restricted datasets in the medical imaging domain might adversely affect model performance. Furthermore, aggravating the problem of model bias and producing less-than-optimal predictions for less-represented classes is the disparity in tumour classifications (e.g., more benign than malignant instances).
- Computational Overhead: Particularly in large-scale MRI datasets, freezing CNN layers during transfer learning lowers the number of parameters to be taught, although the computational demand stays high. The demand for large computing resources, particularly GPU acceleration, could impede the general use of such models in settings with limited resources.
- While CNNs are adept in extracting high-level features, conventional ML classifiers such as Support Vector Machines (SVM) or Random Forests can be prone to overfitting, particularly when considering high-dimensional feature spaces extracted from deep networks. This is especially true in cases when the available training data is either very unbalanced or inadequate, therefore compromising the generalising capacity of the model.
- Lack of transparency and explainability is one of the main obstacles in using deep learning-based models in healthcare environments. Though conventional machine learning classifiers have some interpretability, the hybrid system—which consists of CNN feature extraction followed by ML classification—remains difficult overall. This might make clinical acceptance difficult as patient safety and trust depend on an awareness of the decision-making process.
- Enhanced Model Complexity: Combining CNNs with ML classifiers gives the model design and training procedure even another degree of complexity. Longer training periods and more difficulty fine-tuning hyperparameters—especially in big, complicated datasets—can follow from this. Furthermore, causing questions about model scalability and maintenance in practical healthcare environments is their complexity.

Although the proposed XcepFusion framework achieved high accuracy, there remains a potential risk of overfitting due to certain experimental limitations. Specifically, the use of a single binary-labeled dataset may restrict the model’s ability to generalize to more complex or diverse clinical data. Table 12 summarizes the key sources of potential overfitting and the corresponding mitigation strategies applied in this study.

**Table 12:** Potential risks of overfitting and mitigation Strategies.

Source of Overfitting Risk	Description	Potential impact	Mitigation strategy
Single dataset usage (BR35H)	Model trained and tested on one dataset may not generalize to other data distributions.	Reduced generalization and possible dataset bias.	Future validation on additional datasets (e.g., Figshare, BraTS).
Limited label diversity (binary classification)	Dataset only includes tumor vs. non-tumor categories, lacking multi-class complexity.	Model may not perform well on multi-type tumor detection tasks.	Extend framework to multi-class or multi-modal MRI datasets.
High model capacity	Deep models can memorize patterns instead of learning generalized features.	Overfitting to training data, inflated accuracy.	Applied transfer learning, layer freezing, and pruning to reduce overfitting.
Small sample size	Smaller datasets increase variance and sensitivity to noise.	Unstable model performance across samples.	Use of data augmentation and repeated runs to improve reliability.

## Conclusion

To enhance the accuracy and reliability of MRI-based brain tumor detection, we implemented a hybrid approach that integrates Xception CNNs with traditional machine learning classifiers. By freezing the convolutional layers of the pre-trained CNN, the system efficiently leverages robust feature extraction to obtain high-level representations of medical images without extensive retraining. To address the limitations of both deep learning and conventional approaches, the dense classification head was removed, and the extracted features were fed into classical classifiers such as SVM, KNN, Decision Tree, Random Forest, and Logistic Regression. Our experimental results demonstrate that this combined method achieves strong performance in tumor classification tasks, even with small or unbalanced datasets, while minimizing overfitting and enhancing model stability. Furthermore, the modular architecture allows for clinical personalization and potential integration into decision-support systems. Despite these advantages, the approach has limitations related to data dependency, model complexity, and adaptability to diverse imaging contexts. To further improve model reliability and clinical utility, future work will focus on multi-modal integration (e.g., combining MRI with CT or PET scans) to capture complementary diagnostic information, the incorporation of attention mechanisms and explainable AI (XAI) techniques to enhance interpretability, real-time deployment and clinical testing to evaluate practical performance, and expansion to larger and more diverse datasets to improve generalization across patient populations and imaging conditions. These steps aim to make the framework not only highly accurate but also interpretable, resource-efficient, and clinically applicable for brain tumor detection and diagnosis.

## Data Availability

The dataset used in this study is publicly available on Kaggle under the title BR35H :: Brain Tumor Detection 2020. It can be accessed at the following URL: https://www.kaggle.com/datasets/ahmedhamada0/brain-tumor-detection.
